# GPX4 regulates cellular necrosis and host resistance in *Mycobacterium tuberculosis* infection

**DOI:** 10.1084/jem.20220504

**Published:** 2022-09-07

**Authors:** Eduardo P. Amaral, Taylor W. Foreman, Sivaranjani Namasivayam, Kerry L. Hilligan, Keith D. Kauffman, Caio Cesar Barbosa Bomfim, Diego L. Costa, Beatriz Barreto-Duarte, Clarissa Gurgel-Rocha, Monique Freire Santana, Marcelo Cordeiro-Santos, Elsa Du Bruyn, Catherine Riou, Kate Aberman, Robert John Wilkinson, Daniel L. Barber, Katrin D. Mayer-Barber, Bruno B. Andrade, Alan Sher

**Affiliations:** 1 Immunobiology Section, Laboratory of Parasitic Diseases, National Institute of Allergy and Infectious Disease, National Institutes of Health, Bethesda, MD; 2 T Lymphocyte Biology Section, Laboratory of Parasitic Diseases, National Institute of Allergy and Infectious Disease, National Institutes of Health, Bethesda, MD; 3 Departmento de Bioquímica e Imunologia, Programa de Pós-Graduação em Imunologia Básica e Aplicada, Faculdade de Medicina de Ribeirão Preto, Universidade de São Paulo, Ribeirão Preto, Brazil; 4 Laboratório de Inflamação e Biomarcadores, Instituto Gonçalo Moniz, Fundação Oswaldo Cruz, Salvador, Bahia, Brazil; 5 Multinational Organization Network Sponsoring Translational and Epidemiological Research Initiative, Salvador, Brazil; 6 Curso de Medicina, Universidade Salvador, Laureate Universities, Salvador, Brazil; 7 Department of Pathology, School of Medicine of the Federal University of Bahia, Salvador, Bahia, Brazil; 8 Center for Biotechnology and Cell Therapy, D’Or Institute for Research and Education, Sao Rafael Hospital, Salvador, Bahia, Brazil; 9 Departmento de Ensino e Pesquisa, Fundação Centro de Controle de Oncologia do Estado do Amazonas, Manaus, Brazil; 10 Fundação Medicina Tropical Doutor Heitor Vieira Dourado, Manaus, Brazil; 11 Programa de Pós-Graduação em Medicina Tropical, Universidade do Estado do Amazonas, Manaus, Brazil; 12 Faculdade de Medicina, Universidade Nilton Lins, Manaus, Brazil; 13 Wellcome Centre for Infectious Disease Research in Africa, Institute of Infectious Disease and Molecular Medicine, University of Cape Town, Cape Town, South Africa; 14 The Francis Crick Institute, London, Northwick Park Hospital, Harrow, UK; 15 Department of Infectious Disease, Imperial College London, London, UK; 16 Inflammation and Innate Immunity Unit, Laboratory of Clinical Immunology and Microbiology, National Institute of Allergy and Infectious Diseases, National Institutes of Health, Bethesda, MD; 17 Curso de Medicina, Escola Bahiana de Medicina e Saúde Pública, Salvador, Bahia, Brazil; 18 Faculdade de Medicina, Universidade Federal da Bahia, Salvador, Brazil; 19 Curso de Medicina, Universidade Faculdade de Tecnologia e Ciências, Salvador, Bahia, Brazil; 20 Division of Infectious Diseases, Department of Medicine, Vanderbilt University School of Medicine, Nashville, TN

## Abstract

Cellular necrosis during *Mycobacterium tuberculosis* (Mtb) infection promotes both immunopathology and bacterial dissemination. Glutathione peroxidase-4 (Gpx4) is an enzyme that plays a critical role in preventing iron-dependent lipid peroxidation–mediated cell death (ferroptosis), a process previously implicated in the necrotic pathology seen in Mtb-infected mice. Here, we document altered GPX4 expression, glutathione levels, and lipid peroxidation in patients with active tuberculosis and assess the role of this pathway in mice genetically deficient in or overexpressing Gpx4. We found that Gpx4-deficient mice infected with Mtb display substantially increased lung necrosis and bacterial burdens, while transgenic mice overexpressing the enzyme show decreased bacterial loads and necrosis. Moreover, Gpx4-deficient macrophages exhibited enhanced necrosis upon Mtb infection in vitro, an outcome suppressed by the lipid peroxidation inhibitor, ferrostatin-1. These findings provide support for the role of ferroptosis in Mtb-induced necrosis and implicate the Gpx4/GSH axis as a target for host-directed therapy of tuberculosis.

## Teaser

Tissue necrosis is a major pathophysiological hallmark of disease progression in tuberculosis. In this study, Amaral et al. show that GPX4, an antioxidant selenoenzyme, is an essential regulator of both lipid peroxidation–mediated cellular necrosis and host resistance in *Mycobacterium tuberculosis* infection.

## Graphical Abstract

**Figure:**
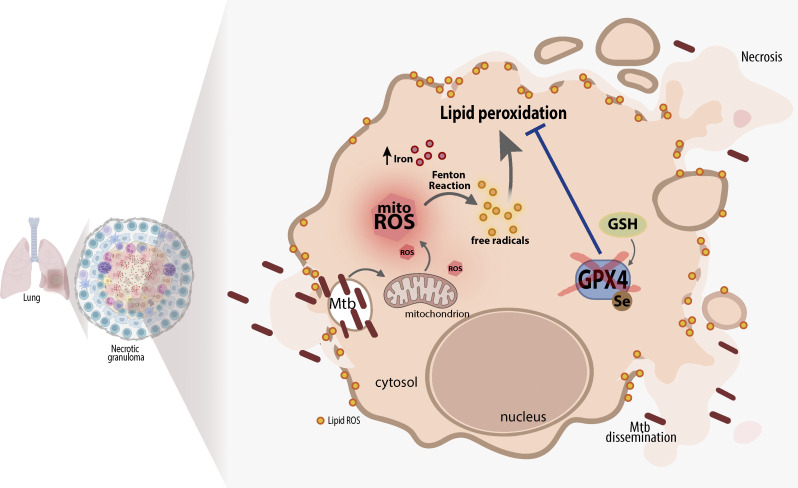

## Introduction

Tuberculosis (TB) induced by *Mycobacterium tuberculosis* (Mtb) remains one of the top 10 causes of mortality worldwide, and was the leading cause from a single infectious agent before the current COVID-19 pandemic (World Health Organization, 2021). Mtb most frequently targets the lungs entering that tissue through infection of alveolar macrophages (AMs). The development of active disease depends on the subsequent spread of the pathogen within other myeloid phagocyte populations (Cohen et al., 2018; O’Garra et al., 2013; Rothchild et al., 2019). Necrotic cell death leading to tissue damage plays a major role in the pathogenesis of TB and has also been implicated as an important cause of pathogen dissemination at both the cellular and organ levels. As such, the pathways responsible for triggering cellular necrosis during Mtb infection represent important targets for host-directed therapeutic (HDT) interventions in TB.

The generation of ROS is one of the initial effector mechanisms deployed by host cells against Mtb infection (Lerner et al., 2015; Marakalala et al., 2016; Matta and Kumar, 2016). Nevertheless, despite the protective effects of this response, the uncontrolled production and accumulation of ROS can be harmful to the host, resulting in cell death and tissue damage (Amaral et al., 2019; Amaral et al., 2021; Shastri et al., 2018; Smulan et al., 2021; Tobin et al., 2010). To prevent this outcome, the host possesses a set of antioxidant pathways that dampen the excessive generation and/or toxic effects of pro-oxidant molecules. A major process by which ROS attacks host cells is through the generation of toxic lipid peroxides that damage biological membranes leading cells to undergo necrotic death. A key pathway for this process is known as ferroptosis because of its involvement with iron metabolism. In a previous study, we showed that Mtb-induced macrophage necrosis is associated with important hallmarks of ferroptosis, including reduced levels of glutathione (GSH) and glutathione peroxidase-4 (Gpx4) along with increased intracellular free iron, mitochondrial superoxide, and lipid peroxidation. Moreover, necrotic cell death in Mtb-infected macrophage cultures was suppressed by both iron chelation and lipid peroxidation inhibition. Additional in vivo experiments revealed that pulmonary necrosis in acutely infected mice is repressed by the inhibition of lipid peroxidation, and this intervention was accompanied by decreased bacterial burden (Amaral et al., 2019). More recent studies employing B6.Sst1^s^ mice that develop pulmonary TB pathology, which more closely resembles that seen in humans, support a critical role for ROS-dependent lipid peroxidation in Mtb-induced cell death. In this work, Brownhill and colleagues showed that bone marrow–derived macrophages (BMDMs) isolated from B6.Sst1^s^ mice display dysregulated antioxidant responses to TNF when compared with normally resistant B6.Sst1^r^ animals. This augmented response was associated with persistent iron- and lipid peroxidation–mediated oxidative damage and enhanced host susceptibility to Mtb infection (Brownhill et al., 2020 *Preprint*). Taken together, the above studies implicated lipid peroxidation as an important mechanism of necrotic cell death and immunopathology in experimental Mtb infection, thus arguing for a role of ferroptosis alongside pyroptosis (Beckwith et al., 2020) and necroptosis (Zhao et al., 2017) in this process. Such involvement is consistent with previous findings documenting high levels of oxidative stress in TB (Amaral et al., 2016; Palanisamy et al., 2011; Rockwood et al., 2017) as well as major roles for iron metabolism in host resistance to the pathogen at the macrophage level (Costa et al., 2016; Phelan et al., 2018; Pisu et al., 2020; Reddy et al., 2018; Rockwood et al., 2017).

Lipid peroxidation has been characterized as a physiological process initiated by the reaction between hydroxyl radicals with polyunsaturated fatty acids in biological membranes, leading to their structural destabilization. To prevent extensive damage to polyunsaturated fatty acid–enriched membranes, host cells generate a specific set of antioxidants that are critical in converting these oxidized lipids from peroxides to a non-toxic alcohol form. Glutathione peroxidases play an essential role in this host antioxidant response. In mammals, the glutathione peroxidase family consists of eight different enzymes distributed in different tissues and cellular compartments (Brigelius-Flohe and Maiorino, 2013). One of these enzymes, Gpx4, a selenoprotein also known as phospholipid hydroperoxide glutathione peroxidase, has been identified as a central regulator of ferroptosis. Utilizing GSH as a cofactor, Gpx4, uniquely among all the glutathione peroxidases, reduces membrane-bound phospholipid hydroperoxides, thus preventing cellular membrane destabilization (Bao et al., 1997; Bao and Williamson, 1997; Ingold et al., 2018; Liang et al., 2009; Sattler et al., 1994; Ursini and Maiorino, 2020; Ursini et al., 1985). Due to its fundamental importance in controlling lipid peroxidation, Gpx4 is essential for murine embryonic development (Yant et al., 2003), and the use of tissue and/or cell-specific conditional knockout animals is thus necessary for investigating the role of this selenoenzyme in vivo and in ex vivo experimental models.

Alterations in both GSH and Gpx4 levels are known to accompany a variety of clinical conditions including TB, and in some cases they are associated with decreased host resistance (Amaral et al., 2016; Guerra et al., 2011; Maiorino et al., 2018; Smulan et al., 2021; Stockwell et al., 2020; Venketaraman et al., 2008; Venketaraman et al., 2006). Diminished levels of GSH have been reported in plasma from TB patients displaying pulmonary disease (Amaral et al., 2016; Venketaraman et al., 2008) as well as in Mtb-infected murine macrophages in vitro (Amaral et al., 2019; Smulan et al., 2021). Furthermore, monocytes/macrophages and T cells from people living with HIV display lowered levels of reduced GSH, and this deficiency is associated with increased susceptibility to Mtb co-infection in ex vivo cultures (de Quay et al., 1992; Eck et al., 1989; Guerra et al., 2011; Morris et al., 2013). Importantly, in these experiments, GSH supplementation improved macrophage and T cell function enhancing the ability of macrophages to control intracellular Mtb growth (Guerra et al., 2011; Morris et al., 2013). Similarly, Gpx4 expression in the T cell compartment plays an essential role in adaptive immunity in murine models of viral and parasitic infections (Matsushita et al., 2015). Less is known about the requirement for Gpx4 in myeloid cell function. In one study, the depletion of Gpx4 in LysM-expressing cells was reported to promote excessive inflammation and tumorigenesis in a murine model of azoxymethane-initiated colon cancer (Canli et al., 2017). In the opposite direction, in several experimental models of inflammatory disease, elevated Gpx4 activity has been associated with host resistance, presumably due to its suppression of tissue inflammation (Chen et al., 2021; Koulajian et al., 2013; Li et al., 2018; Ran et al., 2004b).

In this study, we investigated the association of Gpx4-regulated lipid peroxidation with host resistance and disease in Mtb-infected humans as well as in murine experimental models of pulmonary TB and provided supporting data from tissue staining of the enzyme in lung sections from Mtb-infected non-human primates (NHPs). We show that patients displaying more severe disease exhibit decreased *GPX4* expression in circulating monocytes from peripheral blood mononuclear cells (PBMCs) and reduced GSH as well as increased lipid peroxide levels in plasma. We further demonstrate that reduced *Gpx4* expression is associated with increased necrosis, lipid peroxidation, and bacterial growth, both in vivo and in vitro, while enhanced *Gpx4* expression has the opposite outcome in vivo. Using conditional knockout mice as well as in vitro infection experiments, we reveal that *Gpx4* expression by myeloid cells plays a central role in determining these outcomes. Together, our findings provide further evidence for the detrimental role of Gpx4-regulated necrosis in Mtb infection and identify the Gpx4/GSH antioxidant axis as a candidate target for host-directed therapeutic intervention in tuberculosis.

## Results

### Association of altered GPX4/GSH and lipid peroxidation levels with pulmonary disease in TB patients

**Figure S1.**
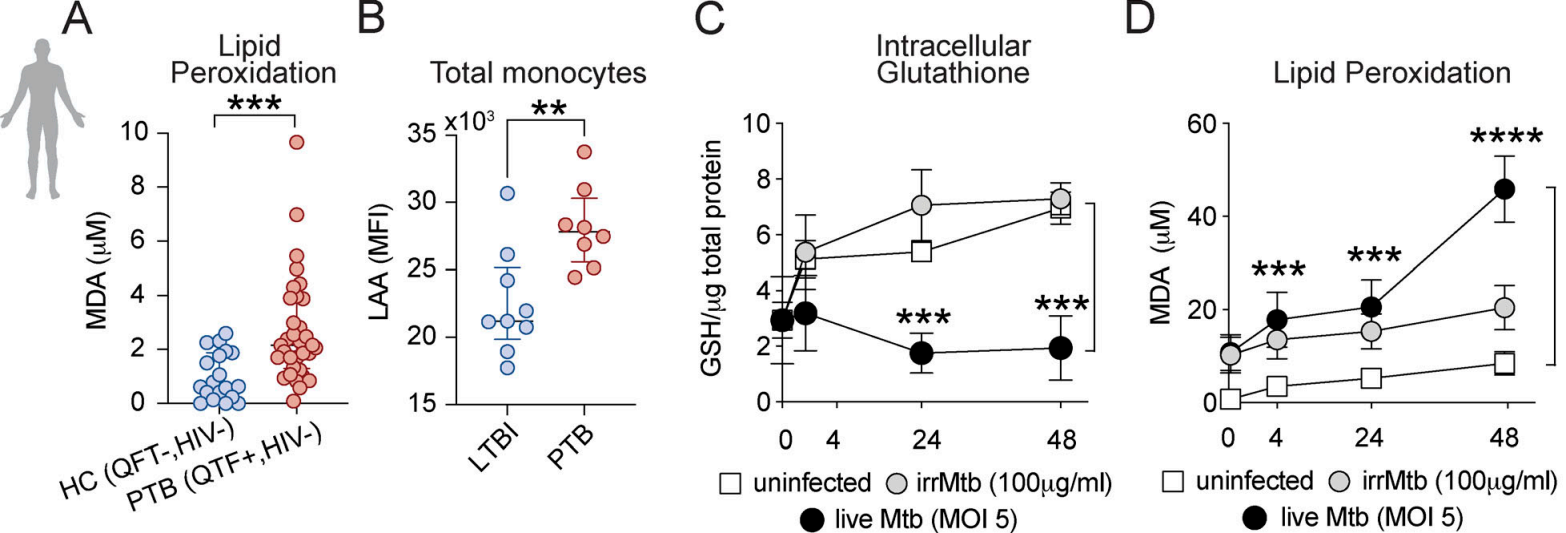
**Evaluation of lipid peroxidation in samples obtained from pulmonary TB patients (South African cohort) and in vitro levels of lipid peroxidation and glutathione in cultures of human monocyte-derived macrophages following Mtb infection. (A)** Lipid peroxidation as measured by MDA levels was assessed in plasma from HC (QuantiFERON-TB [QFT] negative; *n* = 19) and PTB (QFT positive; *n* = 32) HIV negative patients. **(B)** LAA staining in total peripheral blood monocytes (Live/DUMP^-^/HLADR^+^) from LTBI and PTB patients. Each symbol represents an individual patient. **(A and B)** Data represent median values and interquartile ranges. The Mann-Whitney U test was used to compare the significance of the HC versus PTB values or LTBI versus PTB as indicated. **(C and D)** Primary human monocyte-derived macrophages were infected with H37Rv (MOI of 5) or exposed to irradiated Mtb (100 μg/ml). Intracellular GSH (C) and MDA (D) levels were assessed at indicated time-points p.i. as described in the Materials and methods. The data represent means ± SEM of triplicate samples and are representative of three independent experiments. Statistical significance was assessed by one-way ANOVA. Significant differences are indicated with asterisks (**, P < 0.01; ***, P < 0.001; ****, P < 0.0001).

**Figure 1.**
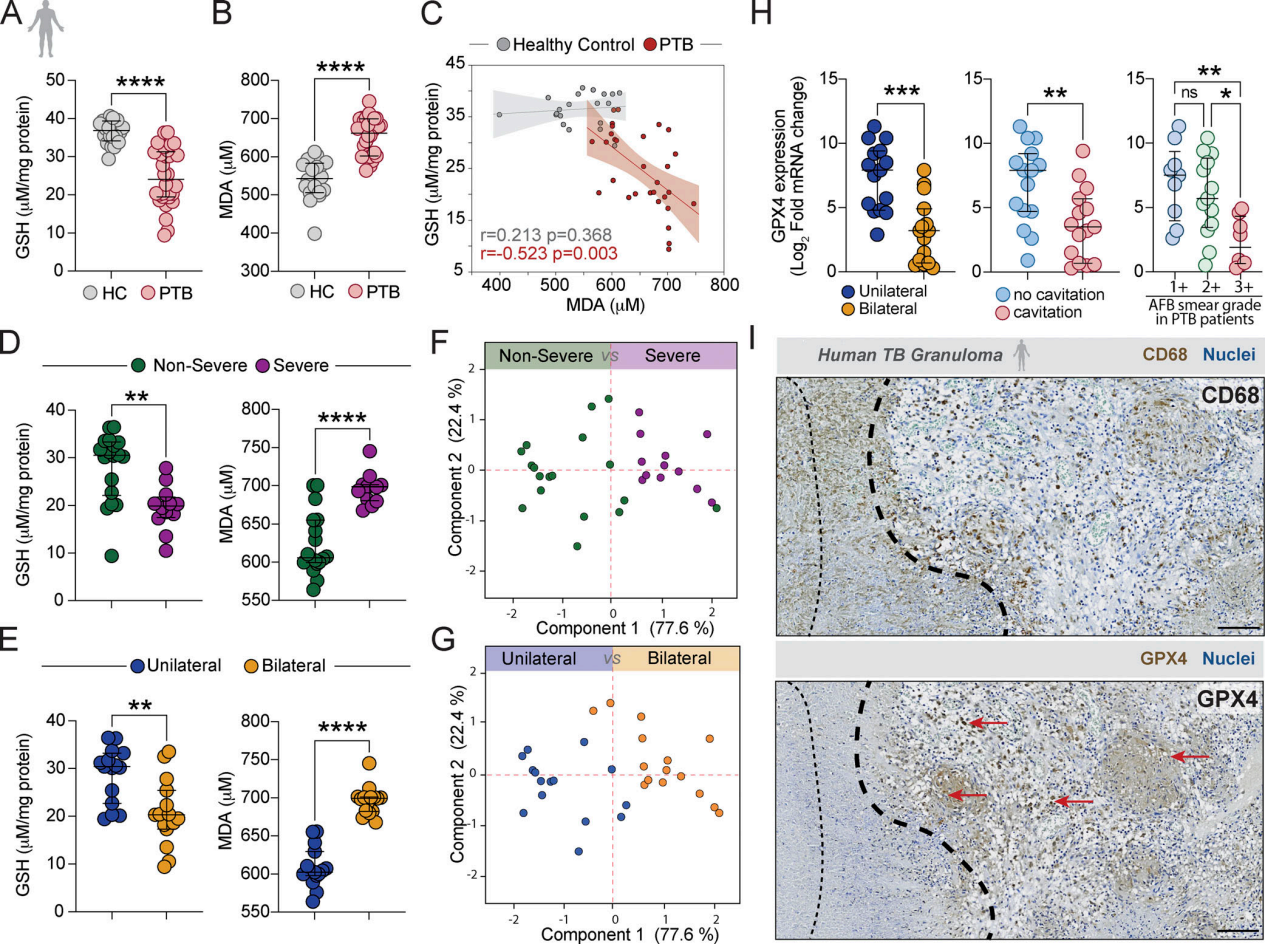
**Dysregulated GSH/GPX4 response and lipid peroxidation in patients with pulmonary TB. (A–G)** Cryopreserved heparinized plasma samples collected from PTB (*n* = 30) and HCs (*n* = 20) from Salvador, Brazil were used in this study. Plasma levels of GSH (A) and lipid peroxidation (MDA; B) were measured using the enzyme-based assays described in the Materials and methods. Data represent median values and interquartile ranges. The Mann–Whitney U test was used to compare the significance of the HC versus PTB values. Each symbol represents an individual subject within the group. **(C)** Correlation between plasma levels of GSH and MDA in HC and PTB for the samples shown in A and B (Spearman correlation test). **(D and E)** GSH and MDA levels in plasma from PTB patients displaying severe (*n* = 12) versus non-severe disease (*n* = 18) as defined in the Materials and methods (D) as well as in subjects with unilateral (*n* = 15) versus bilateral (*n* = 15) lung pathology (E). **(F and G)** Principal component analysis demonstrating three-way association between GSH and MDA levels measured in PTB patients on the basis of disease severity (F) or lung pathology (G). **(H)** *GPX4* mRNA expression in CD14^+^ monocytes isolated from PTB patients and stratified based on lung disease extension, presence of cavitation or AFB smear grade. Data represent median values and interquartile ranges. The Mann–Whitney U test was used to compare values between patient groups. Each symbol represents an individual subject within the group. **(I)** Immunohistochemical staining for CD68 (upper panel) and GPX4 (lower panel) of a post-mortem lung tissue section from a Brazilian PTB patient. The black dashed lines in the image delineate the area surrounding the necrotic core of a granuloma, with the red arrow indicating stronger staining for Gpx4 (scale bars, 100 μm). Statistical significance was assessed by the Mann–Whitney test (for two groups) or Kruskal–Wallis with Dunn’s multiple comparisons or linear trend post-hoc tests (for more than two groups). Significant differences are indicated with asterisks (*, P < 0.05; **, P < 0.01; ***, P < 0.001; ****, P < 0.0001).

As an initial step in evaluating the potential contribution of GPX4 in regulating lipid peroxidation as well as disease in tuberculosis, we analyzed elements of this pathway in TB patients in separate cohorts from Brazil and South Africa. We have previously observed that plasma levels of lipid peroxide products are elevated in Brazilian patients with active disease versus healthy control (HC) subjects (Amaral et al., 2016). We were able to confirm this observation in a separate cohort in Cape Town, South Africa (Du Bruyn et al., 2021; Fig. S1 A) by measuring lipid peroxidation by malondialdehyde (MDA) assay in plasma from pulmonary TB (PTB) and HC individuals as well as by linoleamide alkyne (LAA) staining in total monocytes from latent TB infection (LTBI) and PTB patients (Fig. S1 B). Levels of glutathione, the substrate of Gpx4, are frequently reduced under conditions of ferroptosis (Cao et al., 2019; Dixon et al., 2012; Sun et al., 2018), and this decrease may contribute to the enhanced lipid peroxidation seen in that process. Consistent with this concept and in agreement with previous studies, we observed reduced plasma GSH levels (Fig. 1 A) along with elevated lipid peroxidation (Fig. 1 B) in subjects with PTB versus HC from a different Brazilian cohort. Moreover, further analysis indicated a negative correlation between GSH and MDA levels in the PTB patients, but not in the HC subjects (Fig. 1 C).

We next stratified the Brazilian PTB patients based on their disease severity and lung involvement. This analysis revealed significant differences in glutathione and lipid peroxidation levels between subjects displaying severe (bilateral disease, cavitation, and smear grade ≥2+) versus non-severe disease (unilateral disease, non-cavitation, and acid-fast bacilli [AFB] smear grade <2+; Fig. 1 D) as well as unilateral versus bilateral lung pathology (Fig. 1 E) with decreased levels of glutathione and increased lipid peroxidation correlating with more severe disease. Further, principal component analysis confirmed a three-way association between disease severity as reflected in both clinical parameters and both lipid peroxidation and GSH levels within this patient cohort (Fig. 1, F and G). To experimentally link these observations with Mtb infection, we measured intracellular GSH levels and lipid peroxidation in cultures of human monocyte-derived macrophages exposed to live or irradiated Mtb. The cells exposed to live Mtb showed reduced intracellular GSH along with increased MDA levels (lipid peroxidation) in culture supernatants compared with uninfected cells or macrophages exposed to irradiated Mtb (Fig. S1, C and D). These findings supported an association in human Mtb infection between disease and glutathione-modulated lipid peroxidation, a process known to be biochemically regulated by GPX4.

To directly quantitate *GPX4* expression in PTB patients. we first measured mRNA levels by real-time PCR in purified peripheral blood CD14^+^ monocytes. Individuals in the Brazilian cohort with more severe disease displayed significantly lower levels of *GPX4* mRNA (Fig. 1 H). To evaluate the involvement of GPX4 in TB disease and illustrate it in situ, we examined post-mortem lung tissue sections from Brazilian PTB patients obtained after minimal invasive autopsy and archived as part of the clinical protocol. Following incubation with a polyclonal antibody specific for the enzyme, strong GPX4 staining was observed in cells on the border of granulomas co-localizing with areas positive for CD68, a marker for myeloid cells. In contrast, minimal staining for GPX4 was observed in areas surrounding the necrotic core (Fig. 1 I) consistent with a cytoprotective role for the enzyme.

### Localization of Gpx4 expression in lung tissue from Mtb-infected NHPs and hypernecrotic B6.Sst1^s^ mice

**Figure 2.**
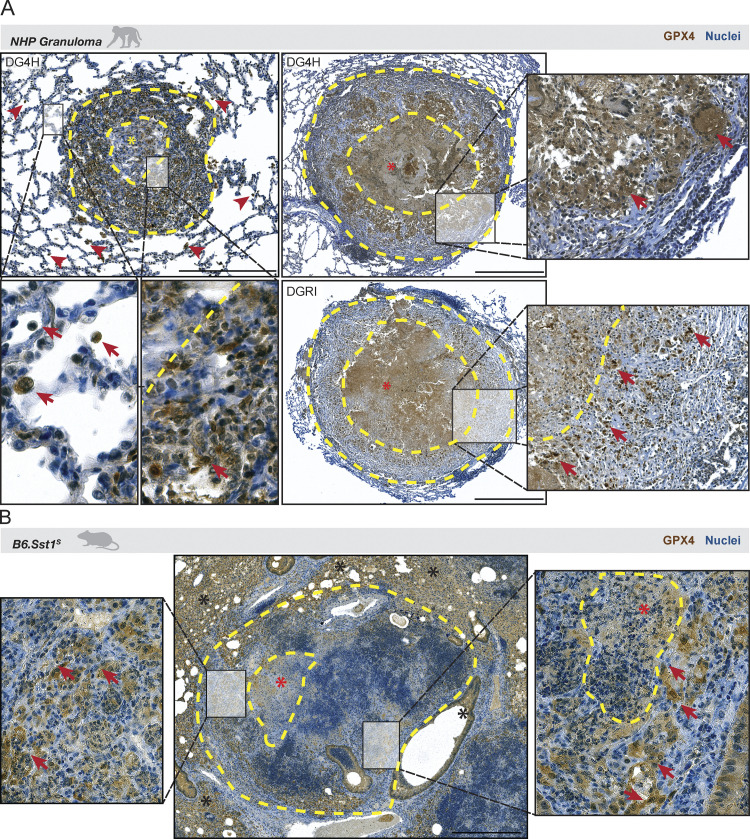
**Spatial distribution of Gpx4 expression in granulomatous lung tissue from Mtb-infected rhesus macaques and B6.Sst1**^**s**^ **mice**. **(A)** Representative pulmonary tissue sections from two rhesus macaques (ID# DG4H and DGRI) infected with H37Rv-mCherry (∼50 CFU) for 16 wk obtained from a previous study (Kauffman et al., 2021) and stained for GPX4. The upper left panel shows a cellular granuloma (delineated by a yellow dashed line), with the myeloid cell enriched center of the granuloma indicated by a yellow asterisk. Red arrows on the upper and lower left panels point out the stronger staining for GPX4 in cells resembling alveolar macrophages compared to other cells. The right panels (upper and bottom) show necrotic granulomas (delineated by a yellow dashed line) from independent animals with the necrotic core outlined by a yellow dashed line and highlighted by a red asterisk (scale bars, 500 μm). Inserts display areas with strong GPX4 staining in live cells as indicated by red arrows. **(B)** Representative lung tissue sections from a B6.Sst1^S^ mouse aerosol infected with H37Rv Mtb strain (∼100 CFU) and euthanized 35 d later. The sections were stained for Gpx4. Yellow dashed lines delineate the granuloma in the middle image, and the red asterisk indicates the necrotic area within the lesion. As shown, strong staining for Gpx4 was observed in cells located at the periphery of the granuloma as well as those situated in the tissue space surrounding this structure. In the right insert, red arrows point out the stronger staining for Gpx4 in live cells that it is absent in areas displaying necrotic cell debris demarcated by a yellow dashed line. The images shown are representative of those observed in five individual animals from two experiments performed (scale bars, 500 μm).

To confirm and extend our observations in TB patients, we also evaluated GPX4 protein expression in lung tissue sections previously obtained from rhesus macaques at 16 wk post-infection (p.i.) with the virulent H37Rv-mCherry Mtb strain (Kauffman et al., 2021). Cellular granulomas isolated from these animals displayed reduced staining for Gpx4 in the center of the granuloma (enriched with myeloid cells) with respect to that observed in cells located on the periphery (cuff; Fig. 2 A, left panel) of these TB lesions. Of note, intense staining of GPX4 was detected in cells resembling alveolar macrophages based on their morphology and location in lung sections from the infected animals (Fig. 2 A, bottom left panel). In addition, necrotic granulomas obtained from these macaques also exhibited stronger GPX4 staining in live cells within the granuloma (Fig. 2 A, right panel).

**Figure S2.**
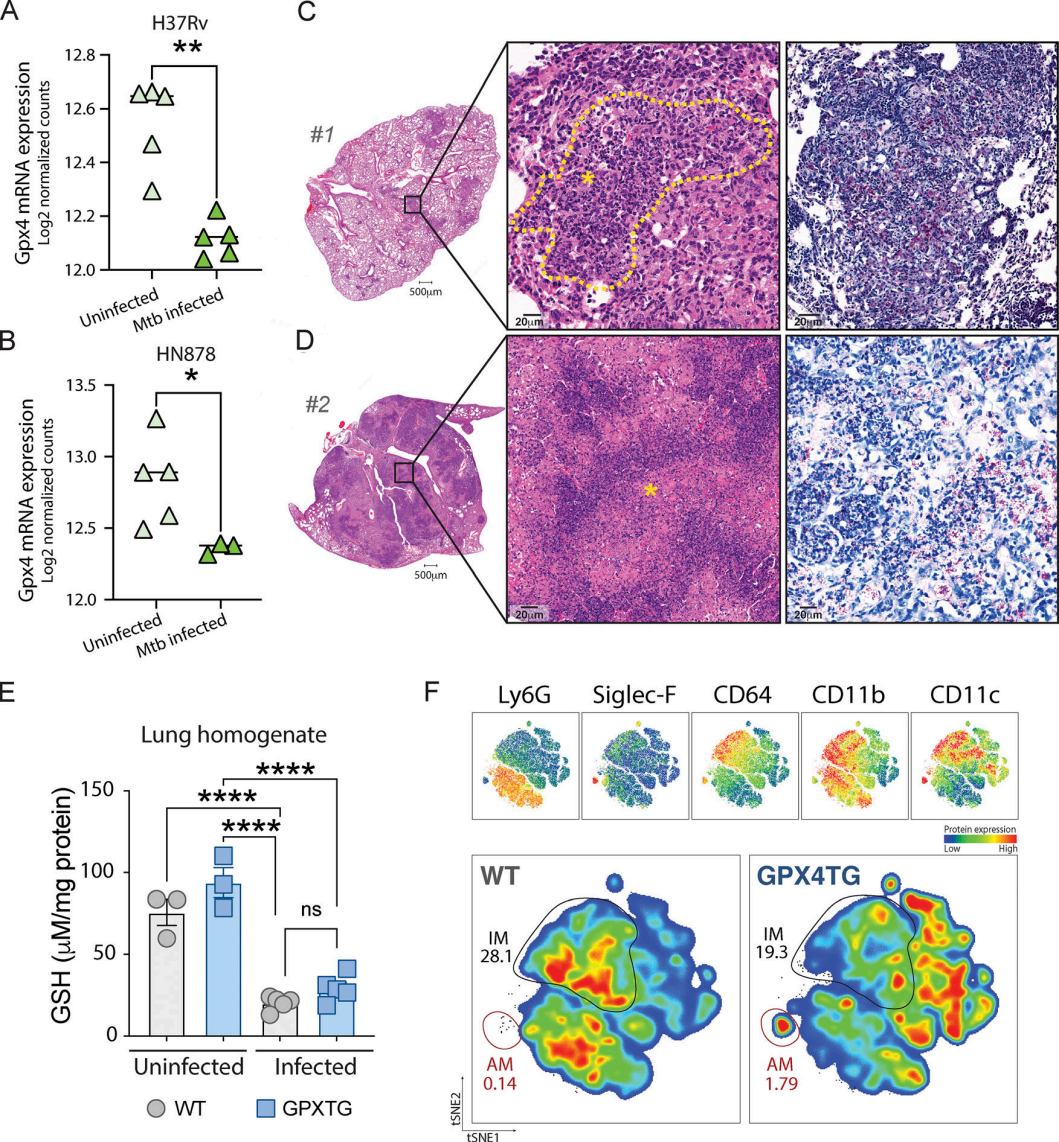
**Gpx4 expression in lungs of C3HeB/FeJ mice, enhanced necrotic cell death in lungs of Mtb-infected cre-ERT2**^**+**^**Gpx4**^**fl/fl**^**, and tSNE analysis of the myeloid compartment as well as glutathione levels in the lungs of GPX4TG mice infected with Mtb. (A and B)** RNAseq data from an available data set previously published by Moreira-Teixeira et al. (2021) were re-analyzed to assess Gpx4 mRNA levels in different experimental settings. In that study, C3HeB/FeJ mice were aerosol infected with a virulent H37Rv Mtb (A) or a hypervirulent clinical isolate HN878 Mtb strain at low dose (∼100 CFU; B) and RNAseq analysis performed in lung homogenates at 42 and 35 d p.i., respectively. Each symbol represents an individual animal within the group. **(C and D)** H&E and ZN staining of lung sections from the two outlier cre-ERT2^+^Gpx4^fl/fl^ mice #1 and #2 shown in the graph on Fig. 3, E and F (scale bars [lower magnification], 500 μm). Lungs from animal #1 in A displayed reduced areas of cellular infiltration compared with mouse #2 in D. Necrotic cell death area is delineated by a yellow dashed line (C) and indicated with yellow asterisks. Numerous AFB (red) are evident in the lungs of both animals (scale bars [higher magnification], 20 μm). **(E and F)** WT and GPX4TG mice were infected as described in the legend of Fig. 3. **(E)** Glutathione levels in lung homogenates from WT and GPX4TG mice at 28 d p.i. were measured. Each symbol represents an individual animal. **(F)** tSNE analysis of the FACS-stained myeloid cells in the lungs of Mtb infected mice. Proportional events from each animal were concatenated (*n* = 5 each group). AMs (red gate) and IMs (black gate) are indicated. Data shown are representative of one of three separate experiments performed. Statistical significance was assessed by the Mann–Whitney test. Significant differences are indicated with asterisks (*, P < 0.05; **, P < 0.01; ****, P < 0.0001).

C57BL/6 mice carrying the sst1-susceptible genotype from C3HeB/FeJ (B6.Sst1^S^ mice) have been previously shown to develop human-like hypernecrotic granuloma lesions following low-dose Mtb infection (Bhattacharya et al., 2021; Harper et al., 2012; Ji et al., 2019). To assess Gpx4 expression with necrotic pathology in this murine model, we aerosol-infected B6.Sst1^S^ mice with ∼100 CFU of the virulent H37Rv Mtb strain and examined lung histology 35 d later. While the overall lung histopathology in this animal strain was distinct from that seen in NHPs and humans, we again observed decreased Gpx4 staining in the central portion of granulomatous lesions and stronger staining for the enzyme in cells located at the periphery as well as in pulmonary tissue areas surrounding the granuloma (Fig. 2 B, left and middle panel). Interestingly, reduced staining for Gpx4 was observed in cells undergoing necrosis within granuloma (Fig. 2 B, right panel). In support of these histopathological findings, analysis of RNA sequencing (RNAseq) data obtained from publicly available data originating from an independent study (Moreira-Teixeira et al., 2020) revealed diminished *Gpx4* mRNA levels in the lungs of Sst1^S+^C3HeB/FeJ mice following aerosol infection with either the virulent H37Rv or the hypervirulent HN878 Mtb strain (Fig. S2, A and B, respectively).

Together, these observations revealed differences in spatial cellular expression of GPX4 in pulmonary granulomatous tissue of experimentally infected animals that would be consistent with a role for this enzyme in the regulation of pathogenesis.

### Mtb-infected mice globally deficient in Gpx4 display increased tissue necrosis while mice overexpressing the enzyme exhibited decreased lung pathology

To assess the functional role of Gpx4 in Mtb-induced disease, we examined the outcome of mycobacterial infection in mice genetically engineered to be either deficient in the enzyme in the whole animal or in specific cellular compartments or to overexpress Gpx4 as a transgene. In the former situation, mice heterozygous for the cre-LOX gene were employed. Thus, complete ablation of Gpx4 protein expression could not be assumed for each animal.

**Figure 3.**
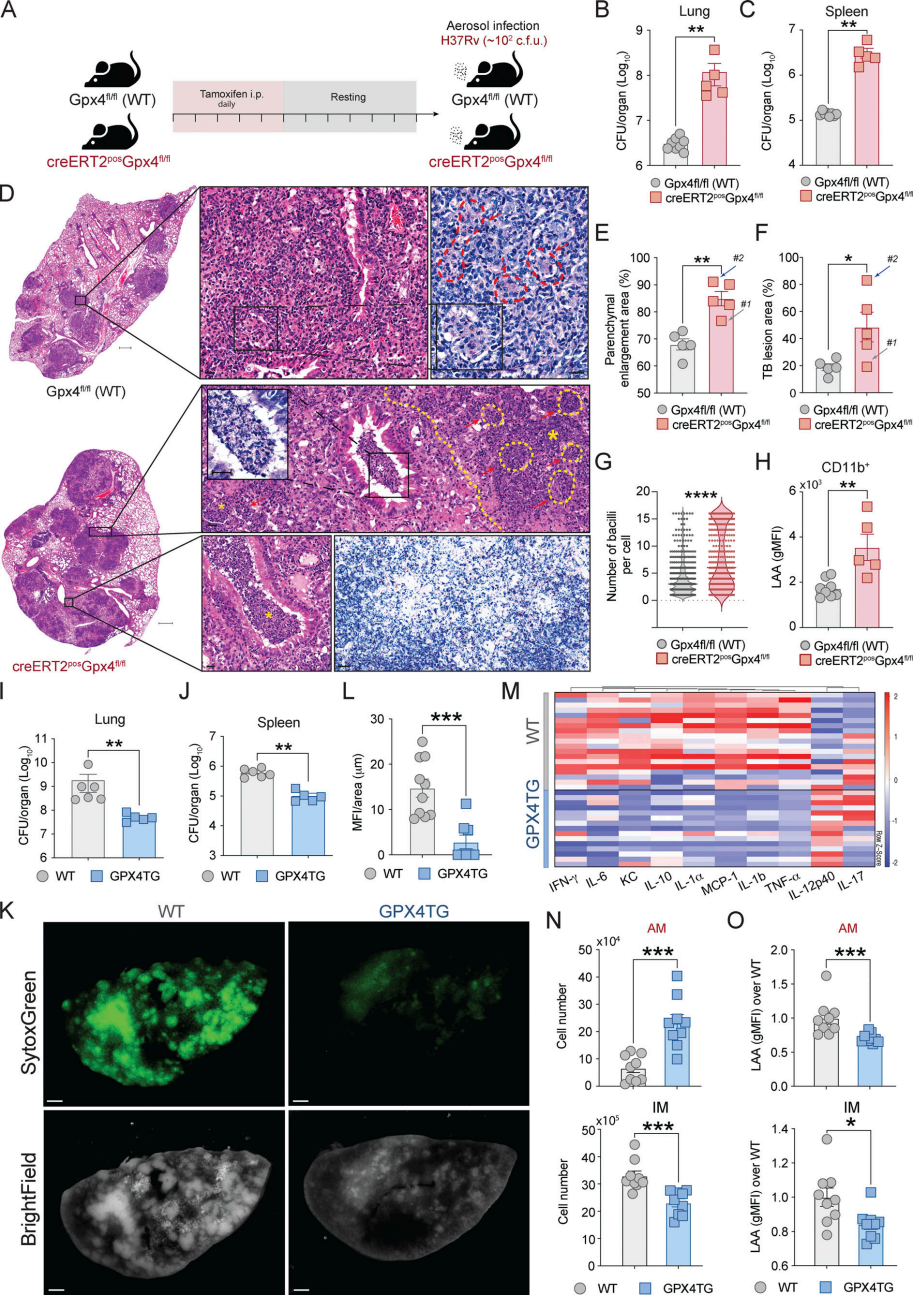
**Alterations in global expression of Gpx4 regulate host resistance to Mtb infection. (A–H)** Gpx4^fl/fl^ (used as control animals) and cre-ERT2^+^Gpx4^fl/fl^ mice treated with tamoxifen were infected by aerosol inoculation with ∼100 bacilli of Mtb (H37Rv). Results are representative of two separate experiments performed for each analysis. **(A)** Scheme of tamoxifen administration. Tamoxifen (2 mg/animal) was given to mice daily via i.p. injections for 5 d. Animals were then rested for 7 d before Mtb infection. **(B–H)** At 28 d p.i., mice were euthanized and lungs and spleens were harvested. Pulmonary (B) and splenic (C) bacterial loads in Mtb-infected mice. **(D)** Representative H&E and ZN images of lungs isolated from Gpx4^fl/fl^ (upper panel) and cre-ERT2^+^Gpx4^fl/fl^ mice (lower panel; scale bars [lower magnification], 500 μm). Each image is representative of tissue sections from five individual mice per experiment. Extensive necrotic lesions (dashed line and asterisk) with intrabronchial accumulation of necrotic cellular material along with elevated numbers of AFB were present in the lungs of cre-ERT2^+^Gpx4^fl/fl^ mice. On the lower ZN panel, wide-spread necrosis with numerous Mtb was evident in the lungs of cre-ERT2^+^Gpx4^fl/fl^ mice (scale bars, 20 μm). **(E and F)** Parenchymal enlargement (E) and TB lesion (F) areas as measured in the lung sections. **(G)** Number of AFB per cell in lung sections assessed by microscopy. **(H)** Lipid peroxidation in live CD11b^+^ cell subset in the lungs analyzed by flow cytometry. **(I–O)** C57BL/6 (WT) and Gpx4-overexpressing (GPX4TG) mice were infected by intrapharyngeal inoculation with ∼1,000–1,500 bacilli of Mtb (H37Rv) as a model of severe TB and the animals sacrificed at 28 d p.i. **(I and J)** Bacterial burdens in the lungs (I) and spleens (J). Data shown are representative of one of five separate experiments performed. **(K)** Lung necrosis evaluated by SytoxGreen DNA staining. **(L)** Mean fluorescence intensity (MFI) of SytoxGreen staining per area of whole lung samples (*n* = 8–10). Data are representative of one of three separate experiments performed (scale bars, 800 μm). **(M)** Heatmap visualization of 10 cytokines measured by multiplex in lung homogenates from WT and GPX4TG mice. Data shown are pooled from three independent experiments. **(N and O)** Cell numbers (N) and lipid peroxidation levels (LAA; O) in AMs (CD45^+^/DUMP^−^/Ly6G^−^/CD24^−^^/low^/IA-IE^+^/CD45iv^neg^/CD64^+^/CD11b^−/low^/CD11c^+^/Siglec-F^+^) and IMs (CD45^+^/DUMP^−^/Ly6G^−^/CD24^−/low^/IA-IE^+^/CD45iv^neg^/CD64^+^/CD11b^hi^/CD11c^−/low^/Siglec-F^−^) analyzed by flow cytometry. The data shown are pooled results from two independent experiments. The data shown in A–O represent the means ± SEM of samples. **(B, C, E–J, and L–O)** Statistical significance was assessed by the Mann–Whitney test and significant differences are indicated with asterisks (*, P < 0.05; **, P < 0.01; ***, P < 0.001; ****, P < 0.0001).

We first studied cre-ERT2^pos^Gpx4^fl/fl^ mice that have been previously reported to exhibit a global reduction in Gpx4 expression in the entire animal (except the brain) upon tamoxifen treatment (Friedmann Angeli et al., 2014; Ingold et al., 2018; Yant et al., 2003; Fig. 3 A). When aerosol-infected (∼100–200 CFU), these mice displayed markedly increased bacterial loads in both the lungs (Fig. 3 B) and spleen (Fig. 3 C) compared with similarly infected non-deficient floxed mice (Gpx4^fl/fl^). Histopathological examination of the lungs of the Gpx4-deficient mice revealed extensive cellular necrosis as evidenced by the presence of large numbers of pyknotic nuclei and cellular debris within bronchial as well as alveolar spaces. In addition, we observed a massive increase in the number of AFB in the extracellular milieu in the lungs of the Gpx4-deficient mice when compared with similarly stained sections from the control animals (Fig. 3 D). Quantitative analysis of the sections revealed a major elevation in the size of the parenchymal areas as well as Mtb-infected granuloma tissue in the Gpx4-deficient lungs (Fig. 3, E and F). While several animals (#1 and #2) within the group appeared to not follow this histopathological pattern, careful examination of their pulmonary lesions nevertheless indicated the presence of necrotic cells as well as numerous associated bacteria (Fig. S2, C and D). Importantly, enumeration of bacilli in all sections revealed a major increase in the number of bacteria per cell in lung of Gpx4-deficient mice versus control animals (Fig. 3 G). Notably, increased numbers of AFB were also observed extracellularly in necrotic areas in the lungs of Gpx4-deficient mice. In addition, flow cytometric analysis demonstrated an increase in lipid peroxidation levels in CD11b^+^ cells as measured by LAA staining (Fig. 3 H) of pulmonary single-cell suspensions from all Gpx4-deficient animals.

In parallel experiments, we examined the effects of Gpx4 overexpression using mice incorporating a *Gpx4* transgene (Ran et al., 2004a). These animals displayed reduced bacterial loads in both lungs (Fig. 3 I) and spleens (Fig. 3 J) compared with WT non-transgenic control mice as well as reduced pulmonary necrosis as measured by SytoxGreen staining (Fig. 3, K and L), a technique that detects available DNA in dying cells together with DNA in the extracellular tissue matrix (Amaral et al., 2019; Marques et al., 2015a). Interestingly, overexpression of Gpx4 in mice did not significantly reverse the suppression of glutathione levels in lung homogenates seen in Mtb-infected WT animals (Fig. S2 E), and this may explain the residual cellular necrosis observed in the lungs of the transgenic mice (Fig. 3, K and L). Multiplex protein analysis of lung tissue homogenates obtained from Gpx4-transgenic mice showed a profound reduction in the levels of many cytokines and chemokines typically associated with the inflammatory response when compared with the homogenates from WT animals (Fig. 3 M). Interestingly, the infected transgenic mice in addition displayed increased numbers of AMs compared with WT animals, suggesting a role for Gpx4 in regulating the fate of these cells during Mtb infection (Fig. 3 N and Fig. S2 F). As predicted, the lungs of the Mtb-infected Gpx4 transgenic mice also showed decreased lipid peroxidation staining in both AM and interstitial macrophages (IMs) as measured by flow cytometry (Fig. 3 O). In addition, the tSNE analysis revealed lowered frequencies of IM and neutrophils in the lung parenchyma of Gpx4-transgenic mice consistent with a general decrease in tissue inflammation (Fig. S2 F).

### Gpx4 deficiency in the myeloid compartment results in decreased host resistance and increased tissue necrosis in Mtb-infected mice

**Figure 4.**
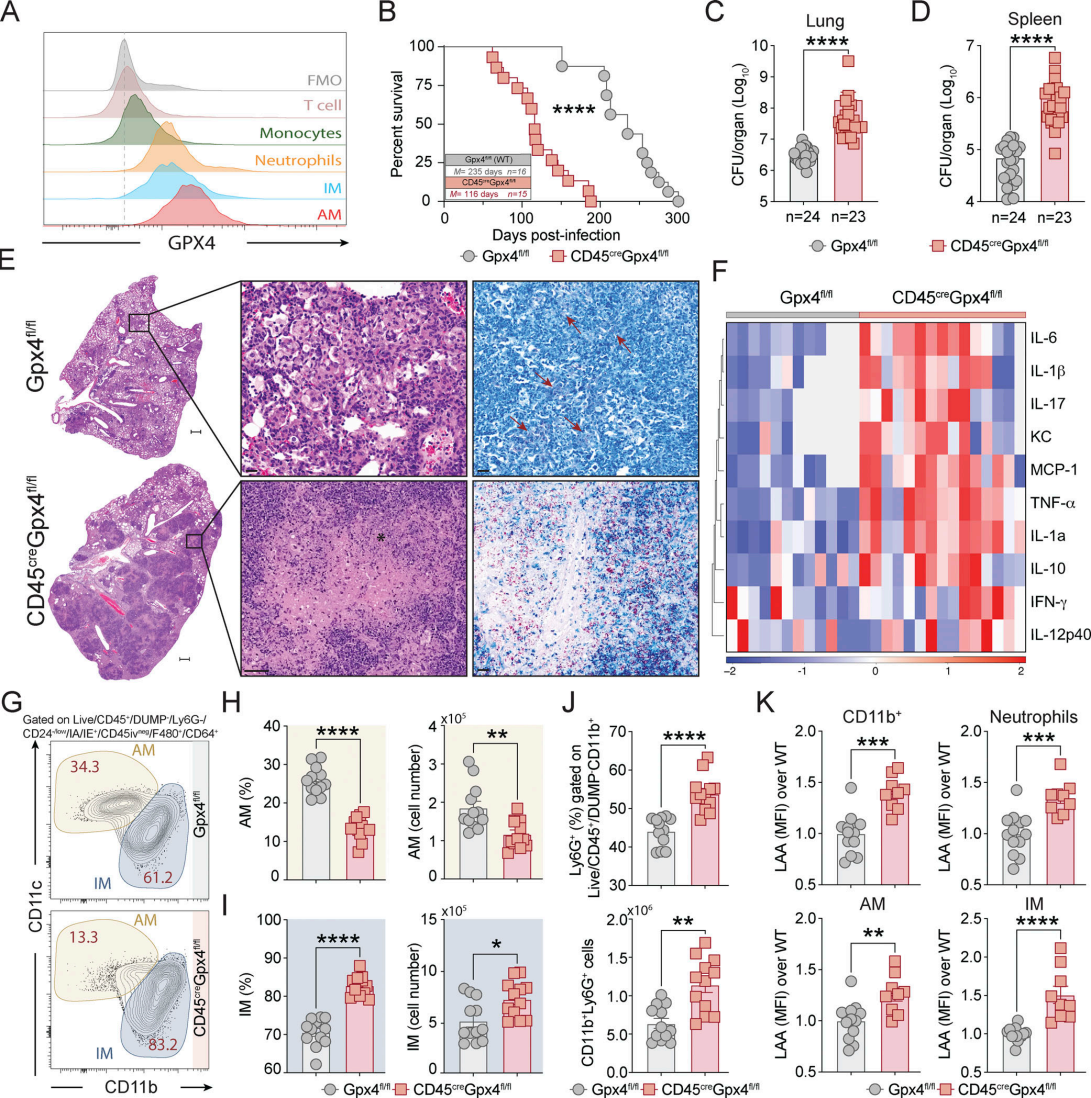
**Deficiency of Gpx4 expression in the hematopoietic compartment enhances host susceptibility to Mtb infection. (A)** Gpx4 expression in different hematopoietic cell types was analyzed by flow cytometry in the lungs of uninfected WT mice. **(B–K)** Gpx4^fl/fl^ and CD45^cre^Gpx4^fl/fl^ mice were infected via aerosol with ∼100–200 bacilli of virulent Mtb H37Rv strain. **(C–K)** Mice were euthanized at 50–55 d p.i. and lungs and spleens were harvested and analyzed. **(B)** Survival curves of Mtb-infected Gpx4^fl/fl^ and CD45^cre^Gpx4^fl/fl^ mice. Statistical significance was assessed by Mantel–Cox test. **(C and D)** Bacterial loads in the lungs (C) and spleens (D) were determined. **(E)** Representative H&E (left panel) and ZN (right panel) images of lungs from Gpx4^fl/fl^ (upper panel) and CD45^cre^Gpx4^fl/fl^ mice (bottom panel; scale bars [lower magnification], 500 μm). Each image is a composite of sections from four to five individual mice in each group per experiment (three independent experiments; scale bars [higher magnification), 20 μm; 50 μm [bottom left panel]). Massive necrotic tissue damage (asterisk) was observed in the lungs of CD45^cre^Gpx4^fl/fl^ mice together with the presence of higher numbers of Mtb (red). Fewer and more isolated AFB (red arrow) were found in the lungs of Mtb-infected Gpx4^fl/fl^ animals. **(F)** Heatmap visualization of 10 cytokines measured in lung homogenates from Gpx4^fl/fl^ and CD45^cre^Gpx4^fl/fl^ mice. Data shown are pooled from four independent experiments (each column represents an individual animal). **(G–K)** Flow cytometric analysis was performed on single-cell suspension from lungs of Mtb-infected Gpx4^fl/fl^ and CD45^cre^Gpx4^fl/fl^ mice. **(G)** Sample FACS plot of parenchymal macrophages. **(H and I)** Summary data of frequency and cell numbers of AM (H) and IM (I). **(J)** Frequency and numbers of Ly6G^+^ cells in the lung. **(K)** Lipid peroxidation (LAA staining) in different myeloid cells in the lungs. Pooled results of three independent experiments are shown (*n* = 12 each group). The data shown in A–O represent the means ± SEM of samples. **(B–D and F–K)** Statistical significance was assessed by the Mann–Whitney test and significant differences are indicated with asterisks (*, P < 0.05; **, P < 0.01; ***, P < 0.001; ****, P < 0.0001).

**Figure S3.**
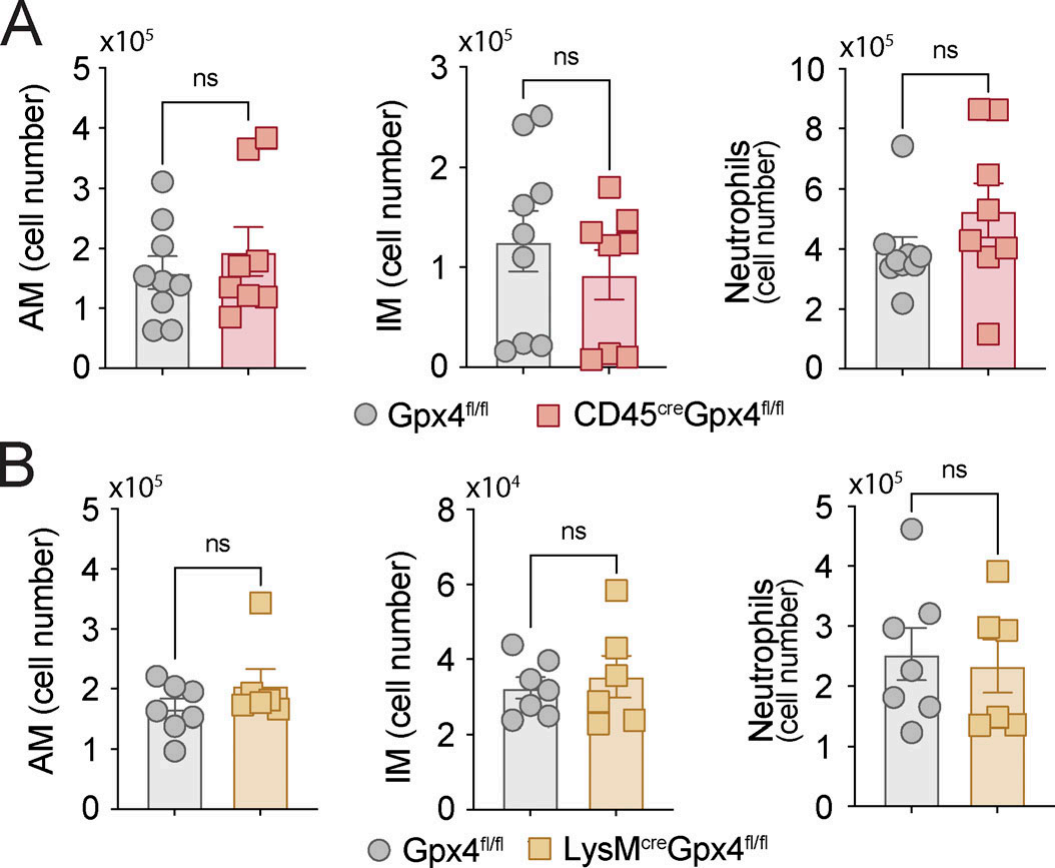
**Gpx4 deficiency does not affect numbers of myeloid cells in the lungs of CD45^cre^Gpx4^fl/fl^, LysM^cre^Gpx4^fl/fl^, and Gpx4^fl/fl^ mice at baseline.** **(A and B)** Total numbers of AMs (Live^+^CD45^+^DUMP^−^Ly6G^−^IA-IE^+^CD24^−/low^CD45iv^neg^CD64^+^CD11c^+^SiglecF^+^), IMs (Live^+^CD45^+^DUMP^−^Ly6G^−^IA-IE^+^CD24^−/low^CD45iv^neg^CD64^+^CD11b^+^SiglecF^−^), and neutrophils (Live^+^CD45^+^DUMP^−^CD11b^+^Ly6G^+^) in the lungs of uninfected Gpx4^fl/fl^ (A and B), CD45^cre^Gpx4^fl/fl^ (A), and LysM^cre^Gpx4^fl/fl^ (B) mice as measured by flow cytometry. Results are pooled from of two independent experiments performed (*n* = 6 each group). Statistical significance was assessed by the Mann-Whitney test.

Gpx4 is expressed in multiple cell lineages within the lung (Fig. 4 A) where it could potentially influence host resistance to Mtb by regulating oxidative stress response to the pathogen (Brigelius-Flohe and Maiorino, 2013). To assess the specific contribution of myeloid cell Gpx4 with respect to other cell populations that produce the enzyme, we examined the outcome of Mtb infection in a series of conditional knockdown mice with genetically engineered Cre-loxP elements to target Gpx4 expression in myeloid cell containing lineages. We first studied CD45^cre^Gpx4^fl/fl^ mice designed to be deficient in Gpx4 expression in the entire hematopoietic compartment (Bohrer et al., 2018; Yang et al., 2008), then LysM^cre^Gpx4^fl/fl^ mice with Gpx4 deficiency targeted to the entire myeloid compartment (Clausen et al., 1999; Nair et al., 2018), and then Mrp8^cre^Gpx4^fl/fl^ mice engineered to be Gpx4 deficient specifically in neutrophils (Nair et al., 2018; Passegue et al., 2004). In preliminary experiments, we determined that depletion of Gpx4 in the myeloid compartment in CD45^cre^Gpx4^fl/fl^ mice and LysM^cre^Gpx4^fl/fl^ mice failed to significantly affect the numbers of AM, IM, and neutrophils at baseline in uninfected animals (Fig. S3, A and B).

**Figure S4.**
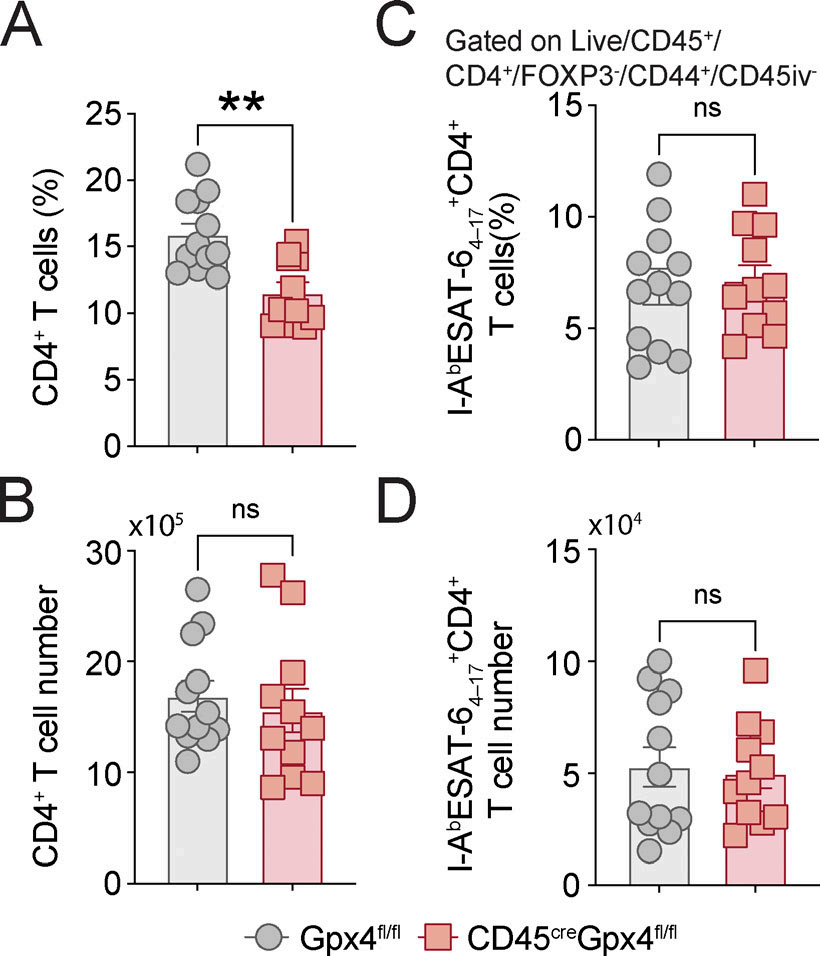
**Gpx4-deficiency does not affect frequency or number of ESAT-6**–**specific CD4**^**+**^ **T cells. (A–D)** Gpx4^fl/fl^ and CD45^cre^Gpx4^fl/fl^ mice were infected via aerosol with ∼100 bacilli of virulent Mtb H37Rv strain and parenchymal CD4^+^ T cells were analyzed at 50–55 d p.i. **(A and B)** Total CD4^+^ T cell frequency (A) and numbers (B) were determined. **(C and D)** I-A^b^ESAT-6_4-17_^+^CD4^+^ T cell frequency (C) and numbers (D) were analyzed. Results are pooled from of three independent experiments performed (*n* = 12 each group). Significant differences are indicated with asterisks and statistical significance was assessed by the Mann–Whitney test (**, P < 0.01).

When infected with H37Rv strain via aerosol (∼100–200 CFU), CD45^cre^Gpx4^fl/fl^ mice exhibited markedly enhanced susceptibility to Mtb, succumbing almost 4 mo earlier than non-Gpx4-deficient floxed control animals (Fig. 4 B). This loss in resistance was associated with greatly elevated bacterial burdens in the lung (Fig. 4 C) and spleen (Fig. 4 D) as well as pronounced pulmonary necrosis (Fig. 4 E) at 45 d p.i., reminiscent of that observed above in the infected cre-ERT2^+^Gpx4^fl/fl^ mice. Consistent with these findings, lung homogenates from Mtb-infected CD45^cre^Gpx4^fl/fl^ mice displayed increased levels of pro-inflammatory cytokines and chemokines with respect to those found in infected non-deficient Gpx4^fl/fl^ animals, with the most prominent changes occurring in the levels of IL-1α, IL-1β, TNF-α, IL-6, and IL-17 along with KC/CXCL1 and MCP-1/CCL2 (Fig. 4 F). Moreover, flow cytometric analysis revealed a pronounced reduction in both the frequency and numbers of AM (Fig. 4, G and H) accompanied by an increase in IM (Fig. 4, G and I) in the lungs of Mtb-infected CD45^cre^Gpx4^fl/fl^ mice. Interestingly, these animals also displayed a preferential enrichment of Ly6G expressing cells in the pulmonary myeloid compartment (Fig. 4 J). While the effect of Gpx4 deficiency in these CD45^cre^Gpx4^fl/fl^ animals is not thought to be unique to myeloid lineage cells, each of the individual myeloid populations studied showed increased lipid peroxidation as measured by LAA staining and analyzed by flow cytometry (Fig. 4 K). Although the Mtb-infected CD45^cre^Gpx4^fl/fl^ mice exhibited a slight reduction in the frequency of CD4^+^ T cells in the lungs (Fig. S4 A), no difference was found in the total numbers of this hematopoietic cell type (Fig. S4 B). More to the point, Gpx4 deficiency in these animals did not affect the frequency and number of Mtb-specific CD4^+^ T cells (Fig. S4, C and D).

**Figure 5.**
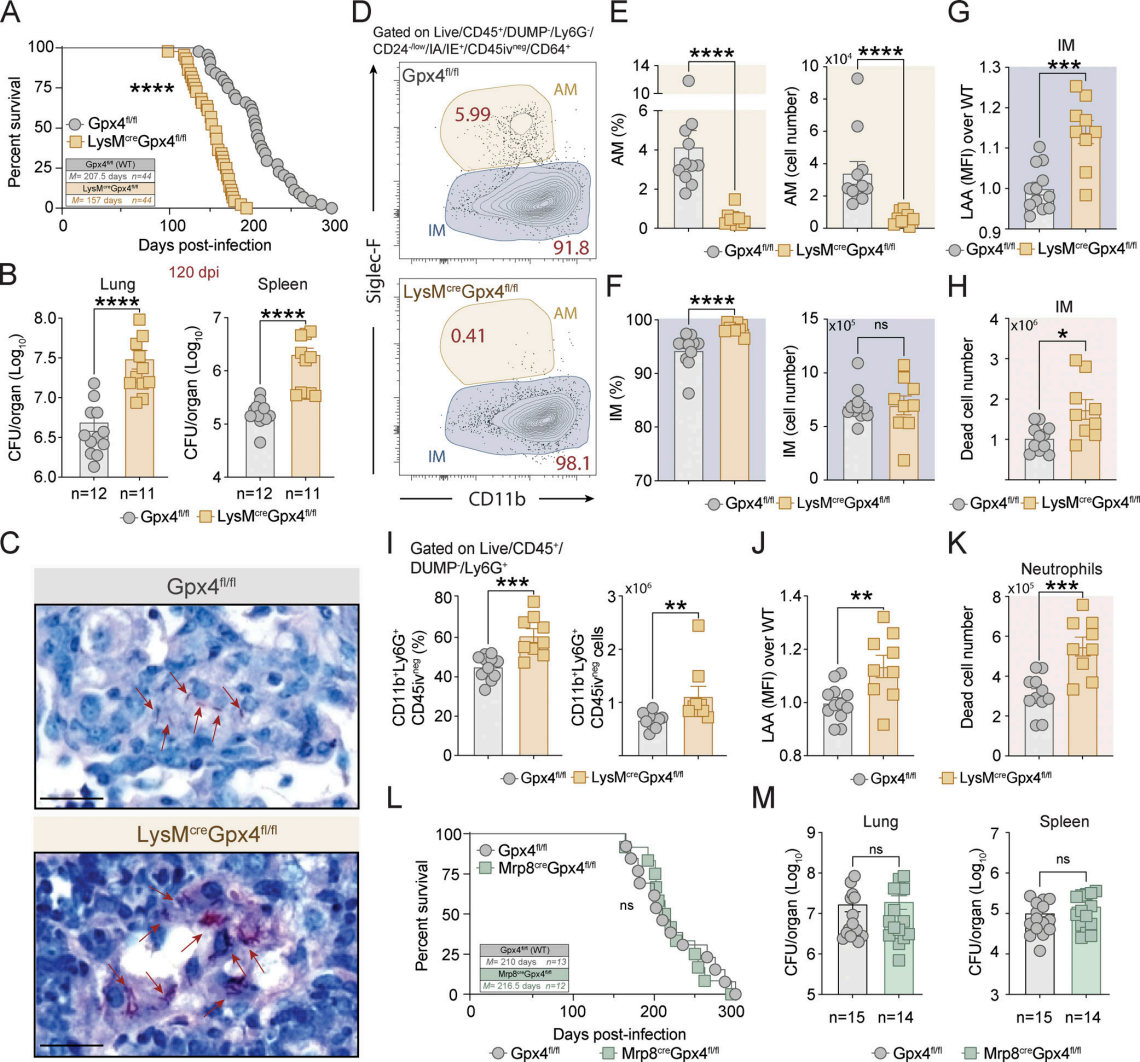
**Gpx4 expression is important for macrophage resistance to Mtb infection in vivo. (A–M)** Gpx4^fl/fl^, LysM^cre^Gpx4^fl/fl^, and Mrp8^cre^Gpx4^fl/fl^ mice were infected via aerosol with ∼100 CFU of virulent Mtb H37Rv. **(B–K)** Mice were euthanized at 120 d p.i. and lungs and spleens were harvested for analysis. **(A)** Survival curves of Mtb-infected Gpx4^fl/fl^ (*n* = 44) and LysM^cre^Gpx4^fl/fl^ mice (*n* = 44). Data were pooled from five experiments. Statistical significance was assessed by Mantel–Cox test. **(B)** CFUs determined at day 120 p.i. by plating lung and spleen homogenates onto 7H11 agar plates. Results are pooled from two separate experiments performed. **(C)** Representative ZN images of lungs from Gpx4^fl/fl^ (left panel) and LysM^cre^Gpx4^fl/fl^ mice (right panel; scale bars, 20 μm). Each image is representative of sections from four to five individual mice per group (two independent experiments). AFB inside macrophages are indicated with red arrows. **(D–H)** Flow cytometric analysis performed on single-cell suspension from lungs of Mtb-infected Gpx4^fl/fl^ and LysM^cre^Gpx4^fl/fl^ mice. **(D)** Sample FACS plot of parenchymal macrophages gated on Live^+^CD45^+^DUMP^−^Ly6G^−^IA-IE^+^CD24^−/low^CD45iv^neg^CD64^+^ events. **(E and F)** Summary data of frequency and cell numbers of AM (E) and IM (F). **(G)** Lipid peroxidation (LAA staining) of the live IM population. **(H)** Numbers of dead IMs. **(I)** Frequency and numbers of Ly6G^+^ cells (neutrophils) in the lung. **(J)** LAA staining of live Ly6G^+^ cells. **(K)** Numbers of dead neutrophils. **(D–H)** Pooled data from two independent experiments were performed (*n* = 9–11 each group). **(L)** Survival curves of Mtb-infected Gpx4^fl/fl^ (*n* = 13) and Mrp8^cre^Gpx4^fl/fl^ mice (*n* = 12). Statistical significance was assessed by Mantel–Cox test. **(M)** Bacterial loads in the lungs and spleens at 30 d p.i. Pooled data from two independent experiments performed (*n* = 14–15). The results shown in A–M are the means ± SEM of data from individual mice within each group. **(B, E–K, and M)** Statistical significance was assessed by the Mann–Whitney test. Significant differences are indicated with asterisks (*, P < 0.05; **, P < 0.01; ***, P < 0.001; ****, P < 0.0001).

We next examined Mtb infection of LysM^cre^Gpx4^fl/fl^ mice that displayed defective Gpx4 expression specifically in the myeloid compartment. LysM^cre^Gpx4^fl/fl^ mice also exhibited increased susceptibility to Mtb infection as evidenced by enhanced mortality and increased pulmonary and splenic bacterial burdens at 120 p.i. (Fig. 5, A and B), although their phenotype was less severe than that seen in CD45^cre^Gpx4^fl/fl^ mice. In addition, histopathology performed on lungs from these animals showed increased numbers of acid-fast bacteria in mononuclear cells (likely macrophages; Fig. 5 C). Flow cytometric analysis performed on single-cell lung suspensions from Mtb-infected LysM^cre^Gpx4^fl/fl^ mice at 120 p.i. revealed a profound depletion in AM (CD45iv^neg^CD64^+^CD11c^+^Siglec-F^+^; Fig. 5, D and E) with no impact on the total numbers of live IM (CD45iv^neg^CD64^+^CD11b^+^Siglec-F^−^; Fig. 5, D and F). Moreover, elevated levels of lipid peroxides were detected in IM from these animals along with increased numbers of dead cells stained with IM markers (Fig. 5, G and H, respectively). Neutrophils were also enriched in the lung parenchyma of Mtb-infected LysM^cre^Gpx4^fl/fl^ mice, and these cells similarly displayed enhanced lipid peroxidation and accumulated as dead cells in the lung parenchyma (Fig. 5, I–K). Nevertheless, the inflammatory phenotype seen in the lungs of Mtb-infected LysM^cre^Gpx4^fl/fl^ mice did not appear to result from an effect of Gpx4 on neutrophils, since Mtb-infected Mrp8^cre^Gpx4^fl/fl^ mice, in which Gpx4 deficiency is specifically targeted to these cells, displayed no detectible loss in host resistance to the pathogen in terms of both survival (Fig. 5 L) and bacterial loads (Fig. 5 M).

Together, these findings demonstrated that the cell-intrinsic expression of Gpx4 in macrophage/monocytes, but not neutrophils, is important for host resistance in experimental Mtb infection.

### Mtb infected BMDMs from mice with myeloid targeted deletions in Gpx4 expression display enhanced ferroptotic death

**Figure 6.**
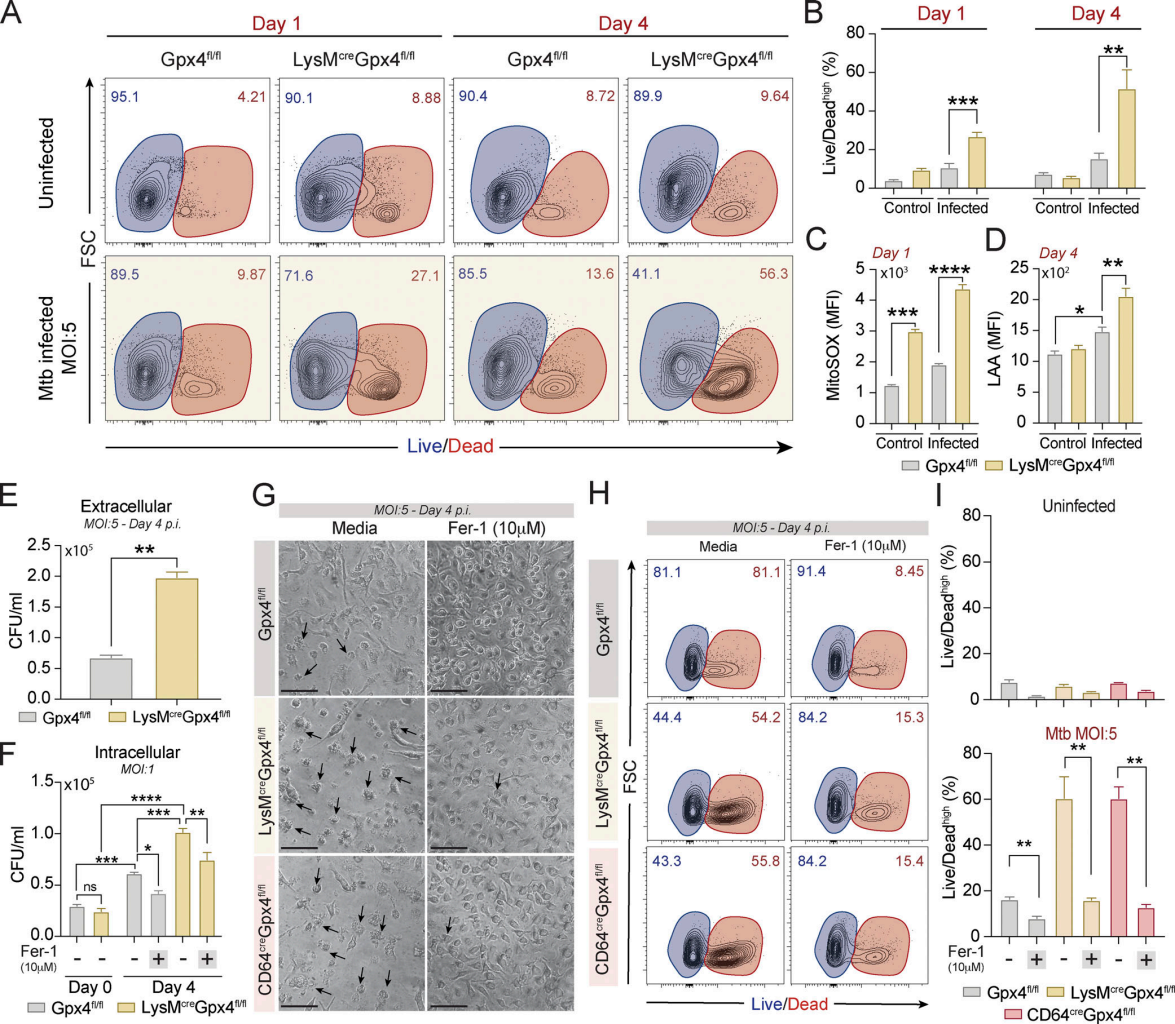
**Gpx4 deficiency Gpx4 triggers lipid peroxidation–dependent necrosis and loss of bacterial control in Mtb-infected macrophages in vitro. (A–I)** Gpx4^fl/fl^, LysM^cre^Gpx4^fl/fl^, and (G–I) CD64^cre^Gpx4^fl/fl^ BMDMs were infected with H37Rv Mtb at different MOIs as indicated. **(A)** Sample FACS plots demonstrating Mtb-induced macrophage necrosis in vitro as measured by Live/Dead staining on the x axis at day 1 versus 4 p.i. **(B)** Summary graph of data shown in A presenting the means ± SEM of triplicate samples analyzed. **(C)** Mitochondrial superoxide was evaluated by MitoSOX staining and analyzed by flow cytometry at 24 h p.i. Results are representative of three separate experiments performed. **(D)** Lipid peroxidation levels in live CD11b^+^ cells were assessed by LAA staining and analyzed by flow cytometry. Representative data from one of at least three independent experiments are shown. **(E)** Extracellular CFU as a readout of bacterial spread was quantified in supernatants from macrophage cultures exposed to Mtb at an MOI of 5. **(F)** Mycobacterial burden evaluated by counting intracellular CFU in macrophage cultures infected at an MOI of 1 on day 0 and 4 p.i. treated or not with Fer-1 (10 μM). Representative data from one of three independent experiments are shown. **(G–I)** Gpx4^fl/fl^, LysM^cre^Gpx4^fl/fl^, and CD64^cre^Gpx4^fl/fl^ BMDMs were infected with H37Rv Mtb at an MOI of 5 and treated or not with the lipid peroxidation inhibitor Fer-1. Necrotic cell death was assessed on day 4 p.i. Results are representative of at least two independent experiments. **(G)** Representative image of Mtb-infected macrophage cultures untreated (left) or treated (right) with Fer-1 (10 μM) on day 4 p.i. (20× magnification; scale bars, 50 μm). Black arrows point out examples of dead cells in the cultures of macrophages treated of not with Fer-1. **(H)** FACS plots of macrophage cultures evaluating cellular necrosis in vitro as measured by Live/Dead staining at day 4 p.i. and analyzed by flow cytometry. **(I)** Summary graph of results shown in H. The data shown in A–I represent the means ± SEM of triplicate samples. Statistical significance was assessed by one-way ANOVA or the Mann–Whitney *t* test analysis for the indicated experimental conditions. Asterisks indicate the statistical differences observed (*, P < 0.05; **, P < 0.01; ***, P < 0.001; ****, P < 0.0001).

**Figure S5.**
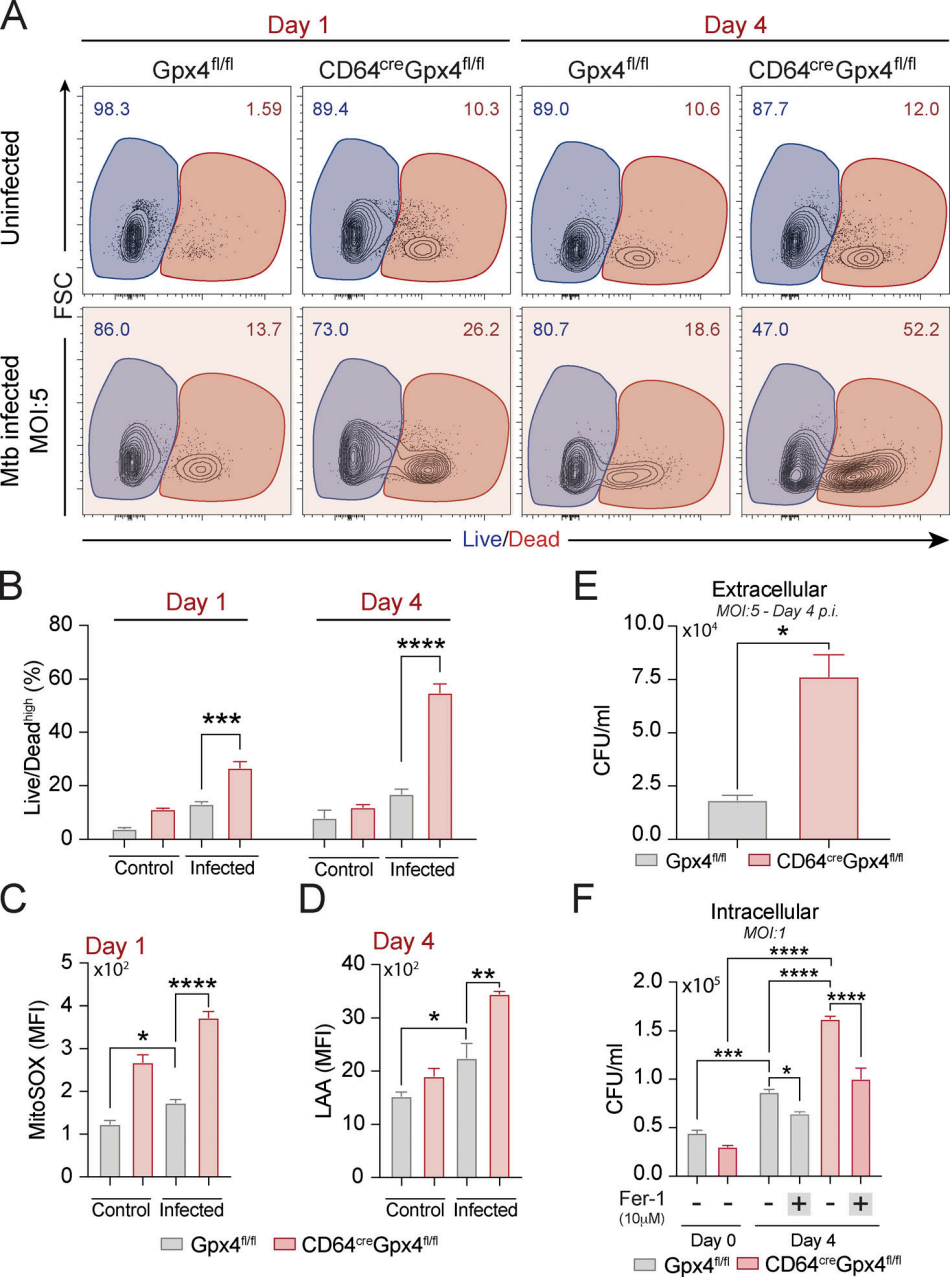
**Ablation of Gpx4 expression in CD64-expressing macrophages increases host cell susceptibility to Mtb infection in vitro. (A–F)** Gpx4^fl/fl^ and CD64^cre^Gpx4^fl/fl^ BMDMs were infected with H37Rv-RFP Mtb at different MOIs as indicated. **(A)** FACS plots demonstrating Mtb-induced macrophages undergoing necrosis in vitro as measured by Live/Dead staining at day 1 versus 4 p.i. **(B)** Summary graph of data shown in A presenting the means ± SEM of triplicate samples analyzed. **(C and D)** Mitochondrial superoxide at 24 h p.i. (C) and lipid peroxidation at 4 d p.i. (D) were evaluated by flow cytometry. **(E)** Extracellular numbers of live Mtb were quantified in supernatants of macrophages infected with H37Rv Mtb at an MOI of 5 on day 4 p.i. **(F)** Intracellular bacterial growth was determined by counting CFU in macrophages infected with H37Rv Mtb at an MOI of 1 on day 0 and 4 p.i. treated or not with Fer-1 (10 μM). The data represent the means ± SEM of samples in triplicate. Statistical significance was assessed by one-way ANOVA or the Mann–Whitney *t* test analysis for the indicated experimental conditions. Results are representative of at least two independent experiments. Asterisks indicate the statistical differences observed (*, P < 0.05; **, P < 0.01; ***, P < 0.001; ****, P < 0.0001).

Gpx4 is known to be a central regulator of ferroptotic cell death (Dixon et al., 2012; Ingold et al., 2018) and was previously implicated by us in the cellular necrosis triggered by Mtb infection in vitro and in vivo (Amaral et al., 2019). To formally test the role of Gpx4 in Mtb-induced cell death, we examined this process in BMDMs from LysM^cre^Gpx4^fl/fl^ and CD64^cre^Gpx4^fl/fl^ mice with targeted deletions in *Gpx4* in myeloid cells. Macrophage cultures from each of these mouse strains when infected at a multiplicity of infection (MOI) of 5 showed enhanced Mtb-induced necrotic cell death at both days 1 and 4 p.i. as measured by flow cytometric analysis (Fig. 6, A and B; and Fig. S5, A and B). This enhanced cellular necrosis was associated with elevated levels of mitochondrial superoxide as well as increased lipid peroxidation (Fig. 6, C and D; and Fig. S5, C and D) and, as expected, resulted in augmented bacterial loads in the extracellular milieu (Fig. 6 E and Fig. S5 E). Interestingly, when examined in cultures infected with Mtb at low MOI (MOI of 1), lack of Gpx4 expression in both conditional knockout macrophage populations was also found to be associated with increased intracellular bacterial growth. This effect was suppressed by treatment with ferrostatin-1 (Fer-1; Fig. 6 F and Fig. S5 F), a specific inhibitor of lipid peroxidation (Dixon et al., 2012; Zilka et al., 2017). Importantly, addition of Fer-1 substantially suppressed the augmented cellular necrosis observed in each of the Gpx4-deficient macrophage cultures infected with Mtb at high MOI (MOI of 5; Fig. 6, G–I). Together, these in vitro observations support a major role for Gpx4 in regulating Mtb-induced macrophage necrosis (ferroptosis) while revealing a potential function for the enzyme in controlling intracellular bacterial growth.

## Discussion

Lipid peroxidation has been implicated as a major cause of necrotic cell death and is thought to contribute to the pathologic consequences of infectious and other diseases (Amaral and Namasivayam, 2021; Fratta Pasini et al., 2021; Jiang et al., 2021; Stockwell et al., 2020). Gpx4, which is expressed by a large number of different cell types, is a central regulator of this process. Here, we show that Gpx4 expression plays a major role in tuberculosis, regulating host resistance to infection as well as tissue pathology, and is required for the survival of Mtb-infected macrophages in vitro. These observations support the targeting of the Gpx4-dependent oxidative stress pathways as a potential host-directed therapeutic approach for intervention in tuberculosis.

Oxidative stress has been long recognized as a pathologic consequence of human Mtb infection (Amaral et al., 2021; Andrade et al., 2013; Pavan Kumar et al., 2013; Rockwood et al., 2017; Trivedi et al., 2012). Here, we demonstrate that patients with more severe tuberculosis display systemic downregulation of *GPX4* expression along with lowered glutathione levels and, as expected, enhanced levels of lipid peroxidation. Interestingly, GPX4 expression was found to be diminished in myeloid cells adjacent to the necrotic core of granulomas in human lung biopsy specimens, suggesting that these cells are in the early stages of necrosis. Further, in vitro exposure of human monocyte-derived macrophages to live, but not dead Mtb, was shown to decrease intracellular levels of glutathione while enhancing lipid peroxidation, confirming this association at the cellular level. Together, the above observations document the contribution of the GPX4/GSH axis to the oxidative stress response triggered during human Mtb infection and disease. Whether this pathway directly plays a direct role in human TB pathogenesis remains to be established. As discussed below, this issue could be further addressed in clinical trials of antioxidant adjunctive therapy.

A major focus of the current study was to test the importance of Gpx4 in host resistance to Mtb infection and necrotic tissue pathology in experimental animal models. In initial experiments in Mtb-infected rhesus macaques, we again observed a reduction in GPX4 expression in cells in the center of the cellular/solid granuloma. Importantly, parallel findings were observed in the B6.Sst1^s^ murine model of Mtb infection that develops human-like hypernecrotic granuloma lesions, demonstrating that similar Gpx4-regulated pathology can arise in mice in addition to primates. With this background information, we addressed whether mice displaying Gpx4 deficiencies are more susceptible to tuberculosis as a consequence of necrotic tissue pathology and the increased bacterial loads that are commonly associated with this outcome (Divangahi et al., 2009; Ehlers and Schaible, 2012).

Impressively, mice designed to be globally deficient in Gpx4 (cre-ERT2^pos^Gpx4^fl/fl^) or with deficiency in the enzyme restricted to the hematopoietic compartment (CD45^cre^Gpx4^fl/fl^) were highly susceptible to low-dose Mtb infection in terms of bacterial loads, pathology, and survival (measured only in the CD45^cre^Gpx4^fl/fl^ animals). As predicted, this loss in host resistance was associated with major increases in lipid peroxidation at the cellular level but was unlinked to any deficit in the production of protective cytokines. Nevertheless, since Gpx4 expression occurs in a variety of cell lineages, it was difficult to attribute this enhanced susceptibility to the events manifested at the level of infected macrophages. In this regard, previous studies have revealed defects in CD4^+^ and CD8^+^ T cell expansion in cre-ERT2^pos^Gpx4^fl/fl^ mice upon tamoxifen treatment that could potentially contribute to the loss in resistance observed here (Matsushita et al., 2015). Nonetheless, the pronounced phenotype of these mice revealed a major role for Gpx4 in regulating host resistance to Mtb infection. This conclusion is consistent with previous data demonstrating increased susceptibility of mice to Mtb infection upon drug-induced GSH deficiency (Cao et al., 2021) and with a number of other studies demonstrating decreased resistance to other infectious agents in mice with genetic or drug-induced defects in glutathione metabolism (Kang et al., 2018; Matsushita et al., 2015; Smulan et al., 2021; Villa et al., 2002). The findings presented here with Mtb infection provide a striking genetic demonstration of the powerful role this pathway can play in protecting the host from the lethal effects of excessive cellular stress. Interestingly, an association has recently been noted between lipid peroxidation and enhanced type I IFN production (Brownhill et al., 2020 *Preprint*; Li et al., 2019), suggesting another mechanism by which glutathione metabolism could influence host resistance to Mtb infection. Furthermore, other components of the redox system, including ferroptosis suppressor protein 1 (FSP1) and thioredoxins, have been shown to influence host cell protection against membrane damage due to oxidative stress (Bersuker et al., 2019; Doll et al., 2019; Muri et al., 2020) and could play an additional role in limiting the effects of Gpx4 deficiency on cell survival in this setting.

Since Mtb primarily infects myeloid cells and can trigger lipid peroxidation–mediated death of infected macrophages (Amaral et al., 2019), a major goal of this study was to evaluate the role of Gpx4 in regulating the myeloid response to Mtb infection. In this regard, Mtb-infected CD45^cre^Gpx4^fl/fl^ mice displayed a pronounced reduction of AM along with an enhanced frequency of pulmonary neutrophils associated with increased lipid peroxidation of these cell types. Nevertheless, it was possible that increased bacterial loads observed in these Gpx4-deficient animals may have contributed to these cellular changes. To more directly address the role of Gpx4 expression in myeloid cells, we studied LysM^cre^Gpx4^fl/fl^ mice in which Gpx4 deficiency is specifically targeted to the myeloid compartment, and that previous studies have shown to display partially reduced Gpx4 expression in dendritic cells, monocytes, AM, peritoneal macrophages, and peritoneal neutrophils (Piattini et al., 2021), while exhibiting no alterations in the frequency of these cell populations at steady state (Canli et al., 2017; Piattini et al., 2021). Following Mtb infection, these animals nevertheless displayed a highly significant increase in pulmonary bacterial burden that, as expected, showed considerable animal-to-animal variation. This outcome was reflected in their decreased survival. However, the increased susceptibility of these LysM^cre^Gpx4^fl/fl^ mice did not reach the level observed with the more extensively defective CD45^cre^Gpx4^fl/fl^ animals, implicating, as expected, additional functions of Gpx4 in host resistance to Mtb due to its expression in other cell lineages. Nevertheless, it could be argued that if Gpx4 expression in Mtb infected myeloid cells is critical to prevent oxidative stress–mediated pathology, these animals should have displayed a more susceptible phenotype. At present, it is difficult to address this issue because of the residual expression of the enzyme in all of the animals studied. In contrast, the failure of Mrp8^cre^Gpx4^fl/fl^ mice to display any loss of resistance argues that neutrophils are not amongst the myeloid cells responsible for the phenotype seen in any of the Gpx4-deficient mice studied here.

A role for myeloid cell–expressed Gpx4 in host resistance to infection and cancer has been documented in many previous studies involving different mechanisms and outcomes (Amaral and Namasivayam, 2021; Canli et al., 2017; He et al., 2022; Kang et al., 2018; Zhu et al., 2019). A protective role for Gpx4 was demonstrated in host resistance to HSV-1, as a consequence of the enzyme preventing lipid peroxidation induced carbonylation of STING, which inhibits its triggering of antiviral type I IFN production (Jia et al., 2020). This outcome was not observed with all viral infections. Gpx4 deficiency in myeloid cells was also shown to increase disease severity in a murine model of polymicrobial sepsis as a result of an enhanced lipid peroxidation–dependent caspase-11 activation and consequently gasdermin D–mediated pyroptosis. The latter effect was reversed by treatment with the antioxidant vitamin E (Kang et al., 2018). In a separate study, LysM^cre^Gpx4^fl/fl^ mice were shown to be more susceptible to azoxymethane-initiated colon cancer by enhancing H_2_O_2_-induced genome-wide DNA mutations in intestinal epithelial cells that stimulated invasive tumor growth (Canli et al., 2017). Nevertheless, in contrast with our findings LysM^cre^Gpx4^fl/fl^ mice showed no defect in host resistance in vivo to *Leishmania major*, a pathogen that similar to Mtb primarily infects macrophages (Piattini et al., 2021). While the basis of this discrepancy is unclear, we speculate that it may relate to the particular importance of the necrotic process in governing the outcome of Mtb infection.

Gpx4 is thought to play a critical role in the regulation of lipid peroxidation–mediated ferroptosis, which we have previously implicated as a mechanism of necrotic cell death and tissue damage in experimental Mtb infection. Consistent with this hypothesis, we observed that BMDMs from LysM^cre^Gpx4^fl/fl^ and CD64^cre^Gpx4^fl/fl^ mice exhibited substantially enhanced necrotic cell death in vitro following mycobacterial infection. This outcome was associated with increased extracellular bacterial release, an expected consequence of cellular necrosis. Importantly, the increased death of the Mtb-infected Gpx4-deficient macrophages was accompanied by elevated lipid peroxidation and was suppressed by the ferroptosis inhibitor, Fer-1. Thus, these findings point to a major role of Gpx4 in the regulation of Mtb-induced cell death and provide important genetic evidence supporting the role of ferroptosis in this process. Since Gpx4-deficient macrophages showed enhanced death as early as 24 h after Mtb infection, a time-point at which only limited bacterial replication is expected (Amaral et al., 2019; Koster et al., 2017; Raffetseder et al., 2014; Stanley et al., 2003), the necrosis observed cannot be directly attributed to increased pathogen growth resulting in mechanical membrane disruption. Nevertheless, the LysM^cre^Gpx4^fl/fl^ and CD64^cre^Gpx4^fl/fl^ macrophages when infected at low dose (MOI of 1) to reveal pathogen growth intracellularly displayed increased bacterial loads at later time-points, and this effect was inhibited by Fer-1 treatment. The latter findings suggest that in addition to its role in ferroptosis, Gpx4-regulated lipid peroxidation may also impact the control of intracellular Mtb growth in infected macrophages.

The results presented here support an important role for the Gpx4-dependent glutathione metabolic pathway in the regulation of Mtb-induced necrosis and implicate this process as a potential target for host-directed therapy of tuberculosis. In this regard, glutathione itself has been shown to regulate Mtb infection outcomes in several murine experimental and clinical TB studies (Cao et al., 2021; Guerra et al., 2011; Morris et al., 2013; Safe et al., 2020a; Safe et al., 2020b; To et al., 2021; Venketaraman et al., 2008; Venketaraman et al., 2006), but to the best of our knowledge glutathione administration, in its reduced form, has never been tested in a formal clinical trial in TB patients. In terms of Gpx4 itself, any HDT specifically targeting this enzyme would have to increase rather than inhibit its activity to be an effective therapy. Since Gpx4 function depends on selenium, one approach would be to provide this micronutrient as a dietary supplement, a general strategy, which in one study involving co-administration with vitamin E was shown to reduce the oxidative stress response while potentiating antioxidant status in patients with pulmonary tuberculosis (Seyedrezazadeh et al., 2008). An alternative strategy would be to target upstream signaling components in glutathione metabolism that positively regulate Gpx4 expression and/or activity. One such component is the nuclear factor (erythroid-derived 2)-like-2 (Nrf-2), which controls the expression of Gpx4 in addition to a variety of other antioxidant molecules. Nrf2 activity itself is governed by the regulatory elements BTB and CNC homology 1 or Kelch-like ECH-associated protein 1, each of which are potential therapeutic targets for controlling Gpx4 expression/function (He et al., 2022; Nishizawa et al., 2022; Padilla and Lee, 2021; Yu and Xiao, 2021). It will be important to assess the role of these Gpx4 regulatory factors in host resistance to Mtb in pursuing this general approach for HDT of tuberculosis.

## Materials and methods

### Clinical study design

This study was conducted according to the principles expressed in the Declaration of Helsinki and approved by the Maternidade Climério de Oliveira Ethics Committee, Federal University of Bahia (protocol number: 037/2011, ethics committee approval number: 034/11). Written informed consent was obtained from all participants. A case–control study of HIV unexposed individuals was performed using cryopreserved PBMC samples and corresponding clinical and epidemiological data obtained from participants enrolled in a translational study performed at the Instituto Brasileiro para Investigação da Tuberculose and at the Hospital Especializado Octavio Mangabeira between December 2015 and January 2018. Markers of oxidative stress and *GPX4* expression were tested in 20 HC individuals who had TB excluded through clinical and radiological investigation and who were IFN-γ release assay (IGRA) negative (tested using the QuantiFERON Gold In Tube [3rd generation; Qiagen]) and 30 with culture-confirmed pulmonary TB. AFB screening in sputum smears (by microscopy) and sputum cultures (Lowenstein–Jensen solid cultures) was performed in all patients (Table S1). TB cases also had a radiographic evaluation and were further stratified based on unilateral versus bilateral lung disease. All the TB cases were enrolled at diagnosis and before the initiation of anti-TB therapy. A volume of 50 ml of venous blood was collected in sodium heparin tubes for isolation of PBMCs from a subset of participants who consented to blood collection. Cells were cryopreserved in liquid nitrogen at the biorepository of the Laboratory of Inflammation and Biomarkers, Fundação Oswaldo Cruz. For the immunological assays performed in the present study, selected samples from individuals with confirmed PTB or controls were matched by age and sex (±5 yr). Sample size was determined based on calculations of study power of 80% (α error, 5%) to detect differences in *GPX4* expression >2% (arbitrary set up) between TB and healthy controls.

The South African patient samples analyzed in this study originated from the Inflammatory Determinants of Tuberculosis Disease Severity study (Du Bruyn et al., 2021), a prospective observational cohort study that recruited adult outpatients with LTBI and PTB from the Site B Clinic Khayelitsha. This study was conducted between March 2017 and December 2019 and was approved by the University of Cape Town Human Research Ethics Committee (UCT HREC 050/2015) and was conducted under National Institute of Allergy and Infectious Diseases (NIAID), Division of Microbiology and Infectious Diseases protocol no.15-0047. Participants with LTBI were healthy, had a positive IGRA (QuantiFERON-TB Gold In-Tube; Qiagen), were sputum Xpert MTB/RIF (Mtb/rifampin resistance mutations; Xpert, Cepheid) assay negative, and had no evidence of TB clinically or on their chest radiographs. All participants in the PTB group tested sputum Xpert MTB/RIF positive and had received no more than 1 dose of anti-tuberculosis treatment at study enrolment. Drug-resistant TB, pregnancy, severe concomitant opportunistic infections, and severe anemia (hemoglobin < 7 g/dl) were exclusion criteria. Sputum Xpert MTB/RIF, sputum culture for Mtb, CD4 count, HIV viral load, and C-reactive protein tests were performed by the National Health Laboratory Services. Blood was collected in sodium heparin tubes and processed within 3 h of collection. PBMCs were isolated by density centrifugation with Ficoll-Paque and cryopreserved until batched extraction for further analysis.

### Laboratory measurements

Glutathione and lipid peroxidation levels were assayed in patient plasma samples using kits from Cayman Chemical following the manufacturer’s protocol. Lipid peroxidation in these samples was assessed by measuring the formation of MDA.

### Cellular assays

Cryopreserved PBMCs were thawed and resuspended in 1640 RPMI medium supplemented with 10% FBS. Monocytes were column purified using CD14 beads and plates at a concentration of 10^6^ cells/ml. RNA extraction was performed using the Qiagen Easy RNA extraction kit. *GPX4* mRNA levels in cells from TB patients and HC subjects were assessed by real-time PCR and gene fold increase relative to β-actin (ACTB) was calculated.

*GPX4* primers: Forward sequence: 5′-ACA​AGA​ACG​GCT​GCG​TGG​TGA​A-3′; Reverse sequence: 3′-GCC​ACA​CAC​TTG​TGG​AGC​TAG​A-5′. *ACTB* primers: Forward sequence: 5′-CAC​CAT​TGG​CAA​TGA​GCG​GTT​C-3′; Reverse sequence: 3′-AGG​TCT​TTG​CGG​ATG​TCC​ACG​T-5′.

### Immunopathological examination of autopsy tissue

All patients were examined using the standard autopsy procedure. Autopsies were performed within 12 h of death and, before the autopsy procedure, the body was embalmed with 10% formalin. Lung fragments selected were fixed for 24 h in 10% neutral buffered formalin, embedded in a paraffin block, 5-μm sections, and submitted to standard processing with H&E staining. Special stains for mycobacteria or fungi were made (Wade for mycobacterium and Grocott-Gomori for fungi).

### Animal experiments

9–12-wk-old male Thy1.1 C57BL/6J mice were obtained through an NIAID supply contract with Taconic Farms and used as WT C57BL/6J controls. *Gpx4*-floxed (JAX# 027964; Jackson Laboratory) mice (Yoo et al., 2012) were purchased from The Jackson Laboratory. These floxed animals were crossed with mice displaying a constitutive expression of Cre recombinase under the control of Rosa-26-cre^ert2^ (*cre-ERT2*^+^, JAX# 008463; Jackson Laboratory; Ventura et al., 2007), S100A8 (Mrp8cre^+^, JAX# 021614; Jackson Laboratory; Passegue et al., 2004), and LysM (*LysMcre*^*+*^, JAX# 00478). Additionally, *Gpx4*-floxed mice were crossed with mice expressing Cre recombinase under the control of CD64 (*CD64cre*^*+*^, B6-Fcgr1-IRES-iCre-TEAL also known as B6-Fcgr1^tm2Ciphe^ or *Fcgr1*-cre; Scott et al., 2018), kindly provided by Dr. Sandrine Henri (Aix Marseille Université, Institut national de la santé et de la recherche médicale, Marseille, France), or CD45 (*CD45cre*^*+*^; Yang et al., 2008), kindly provided by Dr. Alexander Medvinsky (University of Edinburgh, Edinburgh, UK). *GPX4* transgenic breeder mice (GPX4TG^+^) were generously provided by Dr. Qitao Ran (University of Texas Health, San Antonio, TX; Ran et al., 2004a). B6.Sst1^s^ mice were donated by Dr. Igor Kramnik (Boston University, Boston, MA). Automated genotyping of the animals used in this study was performed by Transnetyx using real-time PCR with gene-specific probes. All animal studies were conducted in Assessment and Accreditation of Laboratory Animal Care accredited Biosafety Level 2 and 3 facilities at the NIAID/National Institutes of Health (NIH) using a protocol (LPD-99E) approved by the NIAID Animal Care and Use Committee. Animals were housed under specific pathogen–free conditions with ad libitum access to food and water, and were randomly assigned to experimental groups.

Granuloma containing lung samples isolated from Mtb-infected rhesus macaques (ID#: DG4H and DGRI) initially described by Kauffman et al. (2021) were used in this study. Animals were euthanized 16 wk after infection with ∼50 CFU H37Rv-mCherry. Formalin-fixed paraffin embedded tissue sections from the infected animals were used to assess GPX4 staining in NHP granulomas.

### Bacterial strains and culture conditions

Mtb H37Rv strain was grown in 7H9 broth (Sigma-Aldrich) supplemented with 0.05% Tween 80 (Thermo Fisher Scientific) and 10% oleic acid–albumin–dextrose–catalase (OADC; BD Biosciences) at 37°C. Mtb H37Rv expressing the red fluorescent protein (H37Rv-RFP) was a kind gift from Dr. Joel Ernst (University of California San Francisco, San Francisco, CA) and grown in 7H9 broth (BD Biosciences) supplemented with 0.05% Tween 80 (Thermo Fisher Scientific), 10% OADC, and 30 μg/ml kanamycin (Sigma-Aldrich) at 37°C. Bacteria in mid-log phase (OD 0.6-1.0) were centrifuged at 5,000 rpm for 10 min, resuspended in fresh 7H9 media, and frozen at −80°C in aliquots of ∼10^8^ bacilli/ml.

### Primary cell cultures

Murine BMDMs were generated as previously described (Amaral et al., 2019). Briefly, marrow from both femurs and tibiae was harvested in 1× PBS) and flushed through a syringe with 20G needle. Dispersed cells (3–5 × 10^6^ cells) were then seeded in Petri dishes (100 × 15 mm size) containing 10 ml of BMDM differentiation media (DMEM/F12 containing 2 mM *L*-glutamine [Gibco], 10% FBS, 2% Hepes [Life Technologies], 1 mM sodium pyruvate [Gibco], 25 μg/ml gentamicin [Gibco], and 20% of L929-conditioned media [LCM]). After 4 d of incubation at 37°C with 5% CO_2_, 10 ml BMDM-differentiation media without gentamicin was added. On day 6, macrophages were detached by the addition of cold 1× PBS. The resulting cells displayed 95% macrophage identity phenotypically determined by double positivity for CD11b and F4/80 by flow cytometric analysis. In the experiments performed, BMDM was maintained in Opti-MEM media (Invitrogen) supplemented with 2.5% LCM.

CD14^+^ column-purified human elutriated monocytes obtained from peripheral blood of healthy donors from NIH blood bank under Institutional Review Board–approved protocols of both the NIAID and the Department of Transfusion Medicine were used in this study to generate macrophages in vitro. CD14^+^-purified monocytes were cultured in 96-well plates (Corning) in the presence of RPMI 1640 media containing 10% human AB serum and M-CSF 50 ng/ml (PeproTech) for 7 d. Fresh media containing growth factor (M-CSF) were added every 48 h, as previously described (Amaral et al., 2016; Mayer-Barber et al., 2011). For in vitro experiments, human monocyte-derived macrophages were washed with 1× Dulbecco's PBS (Gibco) and cultured in the Opti-MEM media (Invitrogen) at 37°C in 5% CO_2_ atmosphere. Macrophage cultures were ∼98% pure based on extracellular double staining with anti-CD14 (clone M5E2) and anti-HLA-DR (clone L243) macrophage markers and analyzed by flow cytometry. Lipid peroxidation in culture supernatants was quantified using an assay kit from Cayman Chemical, which detects the formation of MDA. Glutathione levels in cell lysates were measured using kits from Cayman chemical following the manufacturer’s protocol.

### In vivo Mtb infection

Mice were infected by aerosol with the Mtb H37Rv strain at low dose infection (100–250 CFU per mouse) or by intrapharyngeal inoculation of Mtb H37Rv at high dose (1,000–1,500 CFU per mouse). Bacterial intake was determined by plating lung homogenates 2–24 h p.i. on Middlebrook 7H11 agar plates supplemented with 0.5% (vol/vol) glycerol and 10% (vol/vol) OADC enrichment media.

### Single-cell suspension from lungs

Lung lobes obtained from mice were washed with sterile 1× PBS, dissected into small pieces, and then digested in RPMI containing Liberase TL (0.33 mg/ml; Sigma-Aldrich) and DNase I (0.1 mg/ml; Sigma-Aldrich) at 37°C for 45 min under agitation (200 rpm). Enzymatic digestion was stopped by adding FBS. The digested lung tissue was dispersed by passage through a 70-μm pore-size cell strainer. Red blood cells were depleted with ACK lysing buffer (Gibco) at room temperature for 3 min. Cells were washed with 1× PBS supplemented with 10% FBS, centrifuged at 1,500 rpm for 5 min, and the cell pellet was resuspended in RPMI supplemented with 10% FBS. Live cell numbers were enumerated using ViaStain acridine orange propidium iodide staining on a Cellometer Auto 2,000 Cell Counter (Nexcelom). Single-cell suspensions were seeded on a round bottom 96-well plate for further analysis.

### Flow cytometry

Human PBMCs were collected in EDTA and washed in 1× PBS containing 2% FBS and 10% Brilliant Stain Buffer (BD Biosciences), then stained for 30 min at room temperature with the following antibodies: CD66b (clone G10F5), CD192 (clone K036C2), CD56 (clone HCD56), CX3CR1 (clone 2A9-1), CD4 (clone A161A1), CD14 (clone M5E2), CD16 (clone 3G8), CD19 (clone SJ25C1), HLA-DR (clone L243), and CD8 (clone SK1). These antibodies were purchased from Biolegend. Stained cells were then acquired on a LSR II Fortessa flow cytometer and analyzed using FlowJo version 10 software (Three Star).

For the phenotypic analysis of lung infiltrating cells, we distinguished cells within the pulmonary vasculature and lung parenchyma by injecting 2 μg anti-CD45 (clone 30-F11; Invitrogen) intravenously 3 min before euthanasia (Sakai et al., 2014). Cocktails of fluorescently conjugated or unconjugated antibodies diluted in 1× PBS containing 2% FBS and 10% Brilliant Stain Buffer (BD Biosciences) were added to isolated cells and incubated for 30 min at 4°C. Antibodies used were directed against CD11b (clone M1/70), CD11c (clone HL3), Ly6G (clone 1A8), CD24 (clone M1/69), CD19 (clone 1D3), B220 (clone RA3-6B2), CD4 (clone GK1.5 or RM4-5), NK1.1 (clone PK136), CD45 (clone 30-F11), Siglec-F (clone E50-2440), TCR-β chain (clone H57-597), and TCR-γδ (clone GL3), and all were purchased from BD Biosciences; F4/80 (clone BM8), CD45 (clone 30-F11), and FoxP3 (clone FJK-16s) were purchased from Thermo Fisher Scientific; CD8-α (clone 53-6.7), CD11c (clone N418), CD44 (clone IM7), CD45 (clone 30-F11), CD64 (clone X54-5/7.1), CD69 (clone H1.2F3), CD88 (clone 20/70), IA/IE (MHCII, clone M5/114), NK1.1 (clone PK136), and Ly6C (clone HK1.4) were purchased from BioLegend; monoclonal rabbit unconjugated Gpx4 (clone EPNCIR144) was purchased from Abcam. Unconjugated monoclonal rabbit antibody was detected with donkey F(ab′)2 Anti-Rabbit IgG H&L pre-adsorbed (Abcam) and rabbit IgG monoclonal (Abcam) was used as primary isotype control. I-A^b^ESAT-6_4–17_ MHC tetramer was produced by the NIAID Tetramer Core Facility (Emory University, Atlanta, GA). Ultraviolet Fixable Live/Dead cell stain dye was purchased from Molecular Probes-Invitrogen and the staining was performed according to the specifications of the manufacturer. Samples were acquired on a FACSymphony A5 SORP flow cytometer (BD Biosciences) or an LSR II Fortessa flow cytometer and analyzed using FlowJo version 10 software (Three Star).

### Glutathione levels in lungs

Glutathione levels were evaluated in lung homogenates. Briefly, lungs were homogenized in 1× PBS and centrifuged at 12,000 rpm at 4°C for 10 min to remove tissue matrix and cell debris. Supernatants were sterilized by 0.22-μm filtration and stored at −80°C. Reduced glutathione levels were measured by using the Glutathione Assay Kit following the manufacturer’s instructions.

### Multiplex cytokine array

Cytokines in lung tissue homogenates were assessed using a MILLIPLEX MAP Mouse Cytokine/Chemokine Magnetic Bead Panel kit (Millipore Sigma) according to the manufacturer’s instructions and measured using a MAGPIX Instrument (R&D Systems). Total protein was measured by Pierce Protein Assay (Thermo Fisher Scientific) and values were used to normalize cytokine levels based on total protein content.

### Histopathology, immunohistochemistry, and necrotic tissue detection

For histological examination, lungs were fixed with 10% formaldehyde, embedded in paraffin, sectioned, and stained with H&E or Ziehl-Neelsen (ZN). Samples were examined under light microscopy and images were scanned using an Aperio VERSA (Leica Microsystems).

For immunohistochemistry, tissues were collected in 10% neutral-buffered formalin and subsequently transferred to 70% ethanol. Fixed tissue was paraffin embedded, and 10-micron-thick sections were prepared for immunohistochemical analysis. Slides were deparaffinized and treated with AR6 buffer (Akoya Biosciences) for 20 min at 100°C. Slides were next placed in AR9 buffer (Akoya Biosciences) at 100°C and allowed to cool to room temperature for ∼45–50 min. Tissues were then permeabilized using 0.2% TritonX 100 (Millipore Sigma) for 10 min, and non-specific binding blocked by using both FCS and an isotype-matched non-specific immunoglobulin. Primary antibody against GPX4 (EPNCIR144; Abcam) was employed at dilution of 1:600. Following extensive washing, slides were stained with ImmPRESS HRP Horse anti-Rabbit staining kit (Vector Laboratories) and counter-stained with hematoxylin. Slides were then mounted and imaged using an Aperio VERSA (Leica Microsystems).

Immunohistochemistry was performed on human lung tissue as follows: serial sections (5 μm) were prepared from lung samples using standard dewaxing/rehydrating protocols and heat-induced epitope retrieval in a hot water bath (98°C) for 45 min. Endogenous peroxidase blocking (Peroxidase Blocking Solution, Dako) was performed for 10 min, followed by protein blocking with 10% non-fat milk for 30 min. The sections were then incubated with GPX4, and CD68 primary antibodies were incubated for 18 h at 4°C. As a negative control, sections were incubated with the control antibodies with identical isotypes at equal protein concentrations to the primary antibody. After washing with PBS, all sections were incubated with Advance HRP Link for 20 min and then subjected to an additional round of PBS washing followed by incubation with Advance HRP Enzyme (Dako Corporation) for 20 min. Chemical reactions were developed with 3,3-diaminobenzidine (Dako) and all sections were counterstained with Harris hematoxylin.

Slides were scanned using an Aperio digital microscope (Leica Microsystems) and imaged using Aperio Image Scope software (Leica Microsystems). One representative field/sample (200×) was selected by a single experienced pathologist. All immunomarkers were analyzed within these same selected fields.

In vivo necrotic tissue detection in the lungs following high dose Mtb infection was assessed by inoculating mice with a solution of SytoxGreen dye (Thermo Fisher Scientific) at 50 μM intravenously 20 min before mouse euthanasia, as previously described (Amaral et al., 2019). This approach has been used to determine tissue necrosis since SytoxGreen dye stains exposed intracellular and extracellular DNA, which can be visualized by fluorescence microscopy (Marques et al., 2015a; Marques et al., 2015b). After animal euthanasia, lungs were harvested, washed with 1× PBS, and immediately fixed with 10% formaldehyde at 4°C for 48 h. SytoxGreen fluorescence in whole lung tissue was examined in a motorized stereo microscope Leica M205 FA, and images were captured with a CFC345 cooled monochrome camera (Leica) using LAS X (Leica Microsystems) software. Images were processed using Imaris 8.4.1 (Bitplane) software and QuPath (Bankhead et al., 2017) for quantification and visualization.

### Determination of bacterial loads

Bacterial burdens in the lung and spleen homogenates were assessed by serial dilution and plating onto 7H11 agar petri dishes supplemented with 0.5% (vol/vol) glycerol and 10% (vol/vol) OADC enrichment media. Mtb colonies were counted after 21 d of incubation at 37°C.

For in vitro experiments, bacterial counts were determined intracellularly and extracellularly at indicated time points. Extracellular bacteria were evaluated by plating serial dilutions of culture supernatants onto Middlebrook 7H11 agar plates supplemented with 0.5% (vol/vol) glycerol and 10% (vol/vol) OADC enrichment media. Total mycobacterial number (intracellular and extracellular) was evaluated by performing CFU counts of BMDM cultures lysed with 0.1% saponin (Sigma-Aldrich) for 5 min. Mycobacterial colonies were enumerated after 21 d of incubation of 7H11 agar plates at 37°C.

### In vitro macrophage infection

Bacterial aliquots were thawed, diluted in complete 7H9 broth media, and cultured at 37°C for 7 d. Mycobacterial concentration was then assessed by spectrophometry at 600 nm, the cultures centrifuged at 5,000 rpm for 10 min, and resuspended in Opti-MEM. Bacteria were then sonicated for 30 s and homogenized to reduce bacterial clumping.

Macrophages were exposed to either H37Rv or H37Rv-RFP at the indicated MOI for 3–4 h, washed three times with 1× PBS, and then cultured in fresh OptiMEM media. On days 1 and 3, LCM was added to the cultures to a final concentration of 2.5%. In some experiments, Fer-1 (Tocris) was added to the cultures 1 h before infection and maintained in the same media for the entire experiment.

### Measurement of cell death in vitro

Cellular necrosis was evaluated by staining adherent cells with Fixable Viability Dye eFluor780 (eBioscience), according to the manufacturer’s protocol. Briefly, uninfected and Mtb-infected cells were first stained with Live/Dead staining solution (1:500 diluted in 1× PBS) at room temperature for 10 min in the dark and then incubated with anti-CD11b antibody (eBioscience) for an additional 20 min. Cells were next washed with 1× PBS following centrifugation at 1,500 rpm for 5 min and fixed with cytofix/cytoperm buffer (eBioscience) for 1 h at 4°C. Macrophages were then detached, washed, resuspended in 1× PBS with 1% BSA (MP Biomedicals) and analyzed by flow cytometry.

### Mitochondrial superoxide assay in vitro

Cellular mitochondrial superoxide was detected by flow cytometric analysis. Briefly, BMDM cultures were washed with HBSS with calcium and magnesium (Gibco) following centrifugation at 1,500 rpm for 5 min to remove residual culture media. The cells were then stained with 5 μM MitoSOX dye (Life technologies) diluted in HBSS with calcium and magnesium at 37°C for 10 min under light-free conditions according to the manufacturer’s protocol. Additional staining for CD11b was performed by incubating cells with specific antibodies for 30 min at 4°C followed by Live/Dead staining for 10 min at room temperature. The cells were next washed with 1× PBS following centrifugation at 1,500 rpm for 5 min and then fixed with 4% PFA for 1 h at 4°C. After fixation, cells were dislodged, washed, and resuspended in 1% BSA 1× PBS. The fluorescence intensity of mitochondrial superoxide was measured using a flow cytometer. BMDMs incubated with cumene hydroperoxide (200 µM) for 20 min were used as a positive control for mitochondrial superoxide generation.

### Lipid peroxidation assay

Click-iT Lipid Peroxidation Imaging Kit (Life Technologies) was used to assess lipid peroxidation in lung single-cell suspension as well as in macrophage cultures according to the manufacturer’s instructions. Cells were incubated with the LAA reagent (alkyne-modified linoleic acid) for detection of lipid peroxidation–derived protein modifications at 37°C for 1 h and then washed with 1× PBS by centrifugation at 1,500 rpm for 5 min. After centrifugation cells were stained extracellularly with specific antibodies at 4°C for 30 min to determine their phenotype and then incubated with Live/Dead detection reagent as described above. Cellular fixation was performed by adding cytofix/cytoperm (BD Bioscience) for 1 h at 4°C. Fixed cells were washed, resuspended in 1× PBS, and then LAA fluorescence analyzed by flow cytometry. As a positive control for lipid peroxidation, we incubated lung single-cell suspensions or BMDM cultures with cumene hydroperoxide (200 µM) for 1 h at 37°C in the presence of LAA reagent solution.

### Quantification and statistical analysis

#### Statistics

All data in figure legends are presented as mean ± SEM values. For in vivo experiments, the sample size (*n*) and numbers of independent experiments are described in the graphics or provided in the figure legends. For in vitro experiments, the number of experimental replicates is listed in the figure legend. Cytokine levels were normalized to total protein concentration per experiment using GraphPad Prism 9.0 software, and clustered and visualized as a heat map using the R package pheatmap. RNAseq data was obtained from https://ogarra.shinyapps.io/tbtranscriptome/ as referred to in Moreira-Teixeira et al. (2020) and re-analyzed to determine mRNA levels of *Gpx4* in different experimental settings in vivo. Statistical analyses were performed with GraphPad Prism 9.0 software using either unpaired two-tailed *t* test for comparison between two groups or one-way ANOVA for multiple comparisons. The median values with interquartile ranges were used as measures of central tendency and dispersion, respectively, for parameters whose values exhibited a non-Gaussian distribution. The Mann–Whitney test (for two groups) or Kruskal–Wallis with Dunn’s multiple comparisons, or linear trend post-hoc tests (for more than two groups) were used to compare continuous variables. Statistical differences were considered significant when P < 0.05 with asterisks denoting the degree of significance (*, P < 0.05; **, P < 0.01; ***, P < 0.001; ****, P < 0.0001).

### Figure visualization

Figures were generated in Adobe Illustrator and R, incorporating images from BioRender.com.

### Online supplemental material

Fig. S1 shows lipid peroxidation measurements in samples from PTB patients (South African cohort) as well as levels of glutathione and lipid peroxidation in cultures of human monocyte-derived macrophages upon Mtb infection. Fig. S2 shows Gpx4 expression in lungs of C3HeB/FeJ mice, additional histopathological analysis in lungs of Mtb-infected cre-ERT2^+^Gpx4^fl/fl^ and tSNE analysis of the myeloid compartment as well as glutathione levels in the lungs of GPX4TG mice infected with Mtb. Fig. S3 shows enumeration of myeloid cell populations from the lungs of mice deficient in Gpx4 at baseline. Fig. S4 shows quantification of Mtb-specific CD4^+^ T cells in Mtb-infected CD45^cre^Gpx4^fl/fl^ mice. Fig. S5 shows that the depletion of Gpx4 expression in CD64-expressing macrophages enhances cell susceptibility to Mtb infection in vitro. Table S1 contains clinical characteristics of the Brazilian participants.

## Supplementary Material

Table S1 shows clinical characteristics of the Brazilian participants.
