# Supplementary material for: GPX4 regulates cellular necrosis and host resistance in Mycobacterium tuberculosis infection
Source: J Exp Med. 2022 Sep 7;219(11):e20220504. doi: 10.1084/jem.20220504 (PMC9458471; doi:10.1084/jem.20220504)
Supplement: Table S1 — shows clinical characteristics of the Brazilian participants. [file JEM_20220504_TableS1.docx]

**Table S1. Clinical characteristics of the Brazilian participants**

| **Characteristics** | **Healthy controls** | **Active Pulmonary TB** | **P-value** |
| --- | --- | --- | --- |
| N | 20 | 30 |  |
| Age – median (IQR), y | 25 (20-32) | 27 (19-33) | 0.452 |
| Male sex, no. (%) | 10 (50) | 15 (50) | 1 |
| Non-white race, no. (%) | 14 (70) | 23 (76.7) | 0.744 |
| Illicit drug use, no. (%) | 3 (15) | 5 (16.7) | 1 |
| Smoking, no. (%) | 3 (15) | 4 (13.3) | 1 |
| Alcohol use, no. (%) | 5 (25) | 4 (13.3) | 0.454 |
| Prior TB, no. (%) | 0 (0) | 1 (3.3) | 1 |
| Acid-fast bacilli smear grade, no. (%) |  |  | N/A |
| 0 | 20 (100) | 0 (0) |  |
| 1+/scanty | 0 (0) | 9 (30) |  |
| 2 | 0 (0) | 13 (43.3) |  |
| 3 | 0 (0) | 8 (26.7) |  |
